# Biological investigation of resinous endodontic sealers containing calcium hydroxide

**DOI:** 10.1371/journal.pone.0287890

**Published:** 2023-07-17

**Authors:** Carlos Roberto Emerenciano Bueno, Francine Benetti, Marina Tolomei Sandoval Cury, Ana Maria Veiga Vasques, Leopoldo Cosme-Silva, Índia Olinta de Azevedo Queiroz, Ana Cláudia Rodrigues da Silva, Rogério de Castilho Jacinto, Luciano Tavares Angelo Cintra, Eloi Dezan-Junior

**Affiliations:** 1 Department of Preventive and Restorative Dentistry, Endodontic Section, School of Dentistry, São Paulo State University (UNESP), Araçatuba, São Paulo, Brazil; 2 Department of Restorative Dentistry, School of Dentistry, Federal University of Minas Gerais (UFMG), Belo Horizonte, Minas Gerais, Brazil; 3 Department of Restorative Dentistry, School of Dentistry, Federal University of Alagoas (UFAL), Maceió, Alagoas, Brazil; Yerevan State Medical University Named after Mkhitar Heratsi, ARMENIA

## Abstract

The purpose of this study was to evaluate, *in vivo*, the biocompatibility, biomineralization, collagen maturation and the *in vitro* antibacterial and cytotoxicity of resinous endodontic sealers containing calcium hydroxide. Forty rats were implanted with polyethylene tubes containing Sealer 26, Sealer Plus, Dia-ProSeal and an empty tube, examined after 7, 15, 30 and 60 days. Antimicrobial activity was evaluated against *Enterococcus faecalis* by Agar Diffusion Test (ADT) through inhibition zones. For cytotoxicity, undifferentiated pulp cells (OD-21) were cultured and assessed using 3-(4,5-dimethylthiazol-2-yl)-2,5-diphenyltetrazolium bromide (MTT) assay, exposed to dilution of serial extracts at 6, 24, 48h. Cytotoxicity was analyzed by two-way ANOVA and Bonferroni correction. Kruskal-Wallis test followed by Dunn test was performed for nonparametric data (p<0.05). MTT assay revealed cell proliferation affected by sealers extract in all periods (p<0.0001), except for Dia-Proseal and Sealer Plus ⅛ dilution. Subcutaneous analysis showed at day 7^th^ moderate inflammatory infiltration. After 30 days, Sealer 26 still showed moderate inflammatory infiltrate compared to mild inflammation from control and Dia-ProSeal (p = 0.006). At day 60^th^, all groups showed similar mild inflammatory infiltrate (p>0.05). Sealer 26 induced more biomineralization than other sealers in all periods. At 7 and 15 days, all sealers had significant percentage of immature collagen fibers. After 60 days Sealer 26 showed more mature fibers compared to other sealers (p<0.001). All sealers had a smaller zone of inhibition than chlorhexidine, but with no significant difference among any group (p>0.05). All sealers showed satisfactory biological responses with *in vitro*/*in vivo* biocompatibility and antimicrobial activity against planktonic bacteria. Sealer 26 induced more biomineralization than Sealer Plus and Dia-ProSeal.

## Introduction

Endodontic sealers should present characteristics as biocompatibility [1], suitable physico-chemical handling properties [2,3], antibacterial [4] and osteoinductive ability to promote biomineralization [5], preventing reinfection. These features are directly related to the composition of the sealer, since zinc-oxide sealers promote longer period of inflammatory response [6], while sealers containing calcium hydroxide shows enhanced repair [5,7,8].

Endodontic failures may be explained by different factors, but mainly by persistence of infection [9]. *Enterococcus faecalis* is anaerobic gram-positive cocci normally found in the human oral cavity and may adapt to environments with low oxygen levels. Studies showed that *E*. *faecalis* is the most commonly found bacteria in cases of endodontic treatment failure, with a high prevalence of 90% [10]. Even with reduction of bacterial load to levels compatible with periapical healing, these remaining bacteria may occur due to ineffective irrigation, inadequate mechanical preparation with untouched root canal areas or anatomical limitations. Therefore, endodontic sealers with antimicrobial activity may aid to decrease residual microorganisms or prevent their growth [11,12].

Resin-based endodontic sealers containing calcium hydroxide among components are available, aiming to associate the good physical properties of resin sealers with biological features of calcium hydroxide: The Sealer 26^®^ (Dentsply, Maillefer Tulsa, OK, USA) and Sealer Plus^®^ (MK Life, Porto Alegre, RS, Brazil) are both resinous sealer with calcium hydroxide in their composition. Biocompatibility and cytotoxicity of Sealer Plus^®^ was previously demonstrated [1], evidencing superior results than AH Plus^®^ and Endo Fill^®^. Sealer 26^®^ also demonstrated biocompatibility in previous *in vivo* study [13].

The Dia-Proseal^®^ (Diadent, Cheongju, Korea) was released in 2016, also with the proposal of a resin based-sealer containing calcium hydroxide. This root canal sealer showed in early research acceptable physicochemical properties, *in vitro* biocompatibility (cytotoxicity) and sealing ability [14]. However, only in 2019 the sealer was approved by Food & Drug Administration–FDA [15].

Although available in market with proven biocompatibility, neither Sealer Plus^®^ nor Dia-ProSeal^®^ resinous endodontic sealers with calcium hydroxide in composition were tested for *in vivo* biomineralization potential or antimicrobial activity. Also, no *in vivo* tissue response of Dia-Proseal^®^ was found in literature. The biocompatibility test in the subcutaneous tissue of rats is recommended by ISO as primary analysis of new materials, as a quick test to compare groups with a small number of animals [1,5,8].

Thus, the purpose of this study was to evaluated the biocompatibility, biomineralization, cytotoxicity, antibacterial and maturation of collagen fibers of resin-based sealers containing calcium hydroxide Sealer 26^®^, Sealer Plus^®^ and Dia-Proseal^®^ in rats, by subcutaneous inflammatory response, collagen repair, deposition of mineralized tissue and *in vitro* cytotoxicity and antibacterial potential. The null hypothesis was that there are no differences in biological response among these materials.

## Materials and methods

**Fig 1:**
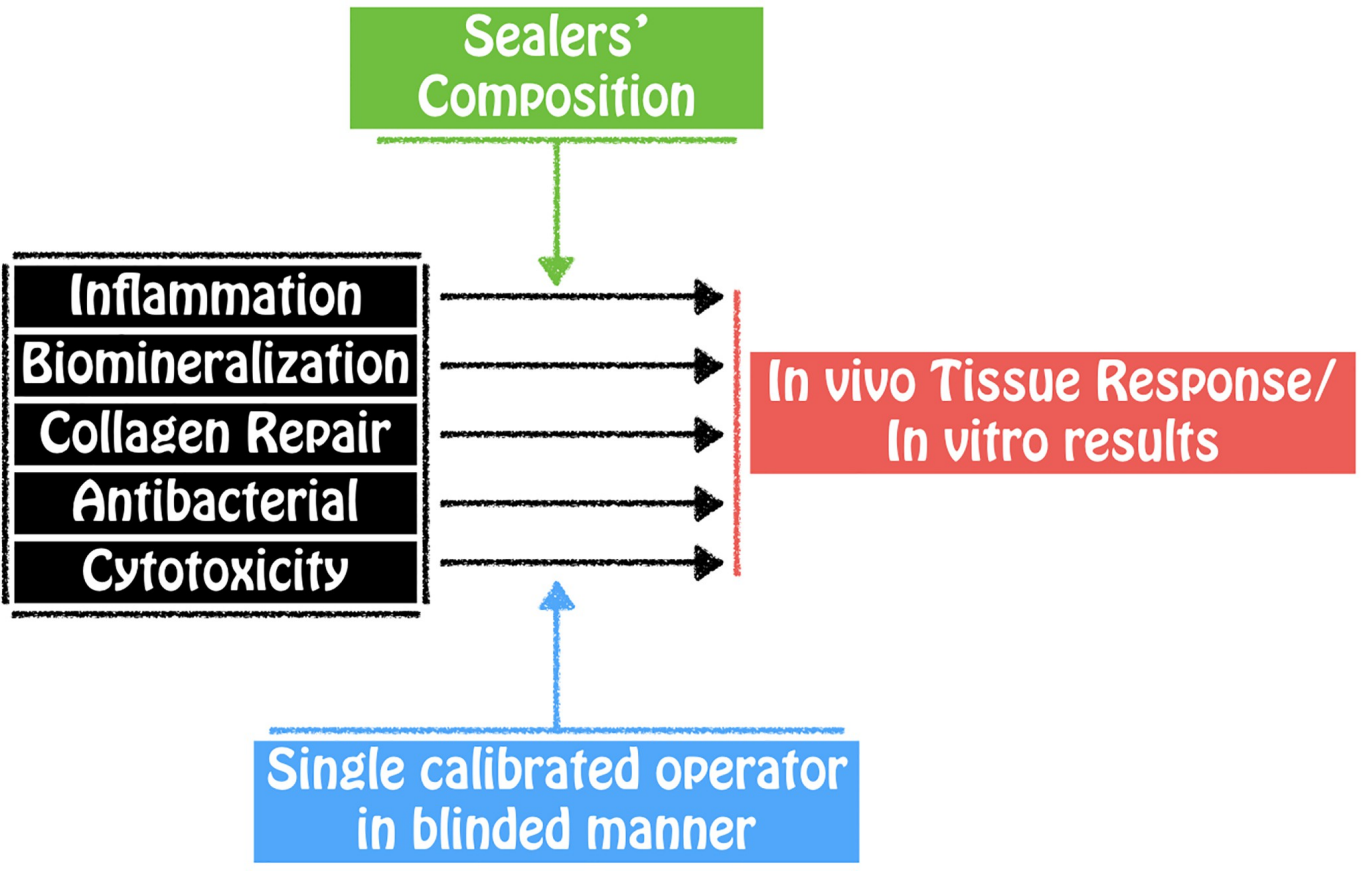
Conceptual framework: Independent variables (analysis) and dependent variables (results). Sealers’ composition was considered as a mediator variable, since differences in composition may affect the dependent variable (results). Single calibrated operator in blind manner was considered a control variable, which aims to maintain the analysis standard.

The conceptualization of the research with its variables and interrelated components is described in Fig 1.

**Fig 2:**
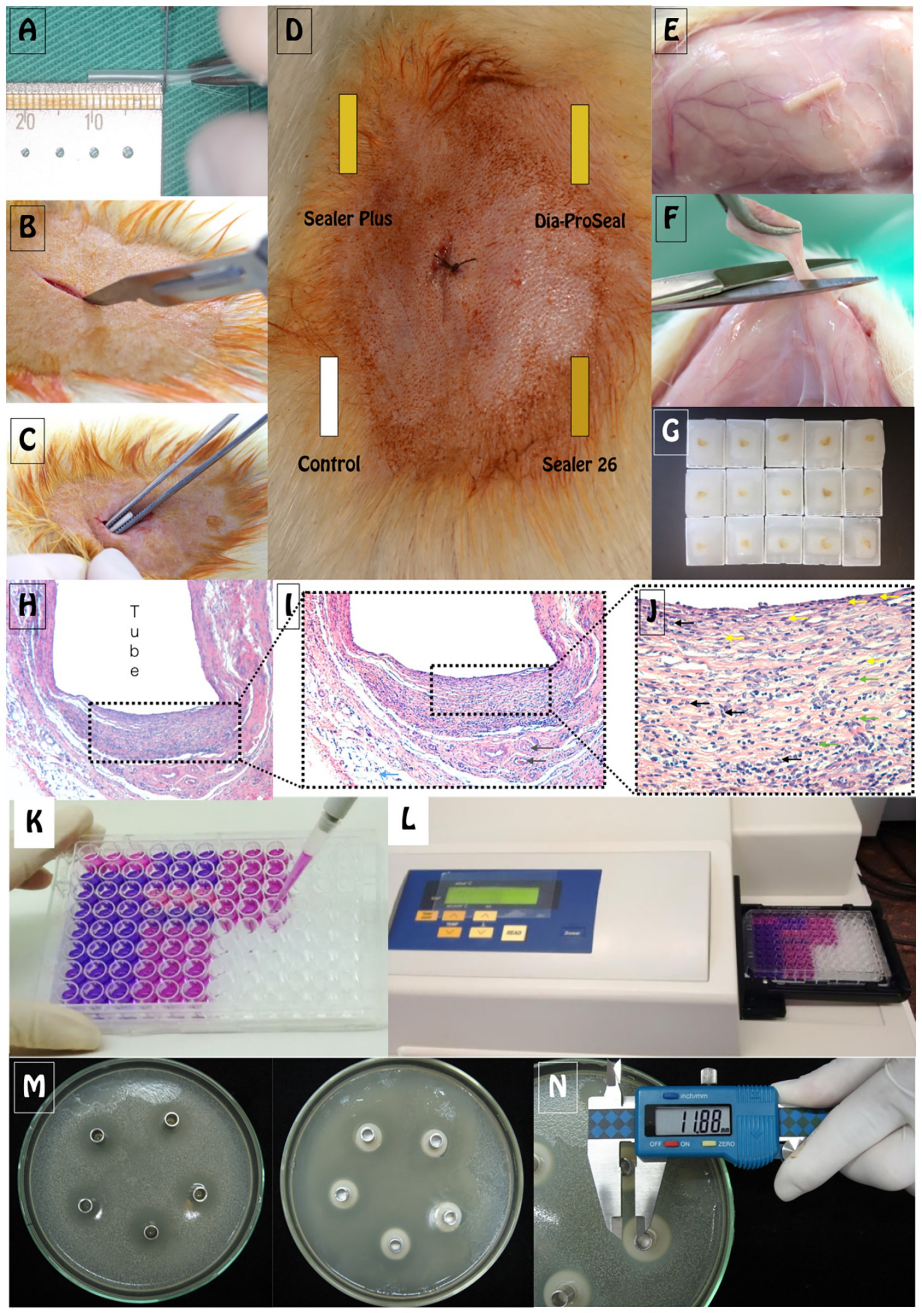
Methodological Illustration: A) standardization of polyethylene tubes for subcutaneous implantation; B) incision in the dorsa of rats; C) implantation of tubes with test material; D) subcutaneous exemplification of tubes location; E) tube exposal after euthanasia; F) tube removal with surrounding tissue; G) after laboratory processing for paraffin embedding; H) area of analysis under optical microscope at tube opening (50x); I) area of analysis at tube opening (100x) with presence of adipose tissue (blue arrow) and blood vessel with red blood cells inside (gray arrow); J) area of analysis (400x) with fibroblasts (yellow arrows), lymphocytes (green arrows) and polymorphonuclear cells (black arrows); K) after dark blue crystals dissolution, the solution was transferred to a 96-well plate to measure the optical density; L) Optical density was measured at 570-nm wavelength using a spectrophotometer; M) petri dish with bacterial suspension and control group (chlorhexidine) or sealer 26 group; N) digital caliper measuring diffusion area (halo).

This study was approved by the institutional Ethics Committee on the Use of Animals at Araçatuba School of Dentistry (CEUA protocol 00326–2018) and conducted in accordance with the ARRIVE (Animal Research: Reporting of in Vivo Experiments) guidelines. All surgery was performed under anesthesia and all efforts were made to minimize suffering. *In vivo* and *in vitro* methodologies are illustrated in Fig 2.

Information in sealer’s composition and means of delivery are described in Table 1. According to each manufacturer, Dia-ProSeal^®^ (Dia-Proseal, Diadent, Cheongju, Korea) and Sealer Plus^®^ (MK Life, Porto Alegre, RS, Brazil) are composed by two pastes: base and catalyst in a proper dual syringe which should be delivered in 1:1 on a mixing pad and mixed with the aid of a spatula until homogenization of a single color. Sealer 26^®^ (Dentsply, Petrópolis, RJ, Brazil) is presented in a powder-paste form and should be manipulated on a thin sheet of glass also with a flexible spatula in a mean proportion of approximately 2 to 3 parts of powder to 1-part resin. The powder must be incorporated to the resin until a smooth and consistent mix is obtained and may be raised with the spatula at a height of 1,5 to 2,5 cm above the glass sheet.

**Table 1:** Tested sealers and composition.

Sealer	Composition	Delivery/Mixture	Manufacturer
Dia-ProSeal	Base Paste (yellow color): 30~35% Diglycidyl ether of Bisphenol A, 30~35% Diglycidyl ether of Bisphenol F, 10~15% zirconium dioxide, 10~15% calcium hydroxide Catalyst Paste (white color): 10~15% Poly(1,4-butanediol)bis(4-aminobenzoate); 15~20% Zirconium Dioxide; 10~15% calcium hydroxide; 25~30% calcium tungstate.	Dual paste-paste syringe/manual	Dia-Proseal, Diadent, Cheongju, Korea
Sealer Plus	Base Paste (yellow color): 40% Bisphenol A-co- epichlorohydrin; Bisphenol F epoxy resin (formaldehyde, oligomeric product with 40% 1-chloro- 2,3-epoxypropanol and phenol); 17% zirconium oxide; 4% silicone and siloxanes; 0.5% iron oxide (pigment); 15% calcium hydroxide. Catalyst Paste (white color): 32% Hexamethylenotetramine; 20% zirconium oxide; 4% silicone and siloxanes; 15% calcium hydroxide; 40% calcium tungstate.	Dual paste-paste syringe/manual	MK Life, Porto Alegre, RS, Brazil
Sealer 26	Powder: 43% Bismuth Trioxide, 37% Calcium hydroxide 14% tetramine hexamethylene and 5% Titanium dioxide. Paste: Epoxy Bisphenol Resin	Powder-paste/manual	Dentsply, Petrópolis, RJ, Brazil

### Cell culture and sealer extract

Murine immortalized cell line: undifferentiated pulp cells (OD-21) were cultured with Dulbecco’s Modified Eagle Medium (DMEM) supplemented by 10% Fetal Bovine Serum (FBS), penicillin, and streptomycin under standard cell culture conditions at 37°C, 100% humidity, 95% air, and 5% Carbon Dioxide (CO_2_).

Dia-Proseal, Sealer Plus and Sealer 26 were mixed according to the manufacturer’s instructions and sealers discs were prepared under aseptic conditions. Briefly, discs were performed using a sterile cylindrical polyethylene tube (diameter: 5 mm; height: 3 mm), kept in incubator at 37°C and 5% CO_2_ for 6h to set and sterilized in ultraviolet light for 1h. Then, discs were incubated for 24h in DMEM supplemented by 10% FBS, penicillin, and streptomycin at 37°C, 100% humidity, 95% air, and 5% CO_2_. The supernatants, referred to as sealer extract, were collected and filtered through a sterile 0.22-mm filter (Sigma-Aldrich, St Louis, MO). Three different dilutions of sealer extract (undiluted, ½ and ¼) were used in this study.

#### Cytotoxicity assay

Cytotoxicity was assessment using 3-(4,5-dimethylthiazol-2-yl)-2,5-diphenyltetrazolium bromide (MTT) assay. Undifferentiated pulp cells (OD-21) were seeded in 96 well-plates at (10^4^cells/well) and incubated for 24h to attachment the cells before addition of sealers extracts. Then, cultures were exposed to serial extracts dilution (undiluted, ½, and ¼). OD-21 cells cultured without extract were used as control. At various time points (6, 24 and 48h) the MTT solution was added to the cell culture and incubated for 4h. Then, MTT solution was then discarded and 200μl of isopropyl alcohol was added to each well and mixed for 30min to dissolve the dark blue crystals. After, blue solution was transferred to new 96-well plate optical density (OD) was measured at 570-nm wavelength. Each condition was analyzed in triplicate (Fig 2).

### In vivo subcutaneous implantation

Forty 3month-old male *Wistar* rats, weighing 250–280 g, were used in the study. The animal sample size was based on previous studies [5,16,17] which used the same methodology to assess biocompatibility and biomineralization in subcutaneous tissue of rats. The animals were housed in temperature-controlled rooms (25 ± 2°C, 70% humidity) and kept under a 12-/12- hour light/dark cycle, with food and water available *ad libitum* for 48 hours before the beginning of the experiment, for an acclimation period.

On hundred and sixty polyethylene tubes (Abbott Laboratories of Brazil, Sao Paulo, SP, Brazil) with a 1.0 mm internal diameter, 1.6 mm external diameter, and 10.0 mm length were used in the study. From this total, forty tubes were separated to be used in control group, as empty tubes. One hundred and twenty polyethylene tubes were filled with the sealers Dia-Proseal^®^ (Diadent, Cheongju, Korea), Sealer Plus^®^ (MK Life, Porto Alegre, Brazil) and Sealer 26^®^ (Dentsply Maillefer, Tulsa, USA), prepared according to the manufacturer’s recommendations and inserted into the tubes with the aid of a lentullo spiral (Dentsply Maillefer, Tulsa, USA).

Anesthesia was obtained with intramuscular administration of xylazine (10 mg/kg Rhobifarma Indústria Farmacêutica Ltda, Hortolândia, Brazil) and ketamine (25 mg/kg União Química Farmacêutica Nacional S/A, São Paulo, Brazil). The backs of the animals were shaved, antisepsis was obtained with 5% iodine solution, and a 2.0 cm incision was formed in a head-tail orientation with #15 Bard-Parker blade (BD, Franklin Lakes, USA), creating two pockets on each side of the incision. Three polyethylene tubes, containing each sealer, and an additional empty tube (control) were implanted in each animal in opposite directions (upper right, upper left, lower right, and lower left) and the skin was closed with a 4–0 nylon suture (Shalon Suturas, Montes Belos, GO, Brazil) (Fig 2).

After 7, 15, 30, and 60 days of implantation, the animals were euthanized by anesthetic overdose. The polyethylene tubes with surrounding tissues were removed (Fig 2) and fixed in 10% buffered formalin at pH 7.0. The specimens were processed for paraffin embedding, serially cut into 5 μm sections, stained with hematoxylin-eosin (HE) and picrosirius red (PSR). The 10 μm sections were stained according to the Von Kossa (VK) technique to observe biomineralization, as it darkly stains mineralized structures or were not stained to be evaluated under Polarized Light (PL) to observe birefrigent structures.

Tissue reactions at the open end of the tubes were scored by a single calibrated operator in blinded manner, according to previous studies [1,5,16–18], as follows: 0, few inflammatory cells or no reaction; 1, less than 25 cells and mild reaction; 2, between 25 and 125 inflammatory cells and moderate reaction; and 3, 125 or more inflammatory cells and severe reaction (400 × magnification). Fibrous capsules were considered thin when < 150 μm and thick when > 150 μm. Calcification was recorded as positive or negative by Von Kossa staining and present or absent under PL (100 × magnification) [5,16,17,19,20].

The maturation levels of the collagen fibers were analyzed in the sections stained by PSR under polarized light microscopy. The program QWin was used (400x magnification; Leica QWin V3; Leica Microsystems), allowing the selection of corresponding colors for each type of collagen fiber in the subcutaneous tissue. After color selection, the program automatically calculated the marked area of each collagen type inside the determined area. Greenish-yellow fibers are considered immature and thin, whilst yellowish-red fibers are considered mature and thick [17].

### Antimicrobial activity assay: Agar well diffusion test

The microbiological assays were carried out under aseptic conditions in a laminar flow chamber. The antibacterial activity was evaluated using a standard strain of *Enterococcus faecalis* (ATCC 51299). The microorganisms were cultivated in Brain Heart Infusion (BHI) broth (Merck, Darmstadt, Germany) at 37°C for 18 h.

A bacterial suspension was obtained with 0.85% saline solution to match the turbidity equivalent to 0.5 McFarland standard tube, corresponding to 1.5 x 10^8^ CFU (Colony Forming Unit)/mL. Six replica plates containing Brain Heart Infusion (BHI) agar (Difco Lab., Detroit, MI, USA) were spread with 0.1 mL of the bacterial suspension with the aid of a Drigalsky’s loop. Then, four wells of 6 mm in diameter and 4 mm in depth (one for each material) were made with a punch by removing the agar at equidistant points and immediately filled with the three sealers (Dia-ProSeal^®^, Sealer Plus^®^, and Sealer 26^®^) to be evaluated and chlorhexidine as positive control [21]. Two plates did not receive the bacterial suspension: one did not receive the sealers aiming to control sterilization of the culture medium; the other plate received the sealers and aimed to control their contamination. All plates were kept at room temperature (22°C ±1) for 2 h for prediffusion of materials and then incubated at 37°C for 48 h under aerobic conditions.

After experimental period, each one of the wells had their inhibition zones measured by the same operator in two perpendicular locations with a sliding digital caliper (Mitutoyo, Absolute Digimatic, Japan) (Fig 2). The size of the inhibition zone was calculated as follows [22]: size of inhibition zone = (diameter of halo–diameter of specimen) x 1⁄2. All the assays were conducted in triplicate and the results were recorded in terms of the average diameter of inhibition zone.

### Statistical analysis

Data was analyzed using Sigma Plot software 12.0 (Systat Software Inc., CA). *In vitro* cytotoxicity data was analyzed by two-way ANOVA followed by the Bonferroni correction. The Kruskal-Wallis followed by the Dunn test was performed to analyze biocompatibility data. Microbiology results were submitted to Kruskal-Wallis test and Picrosirius Red values analyzed by one-way ANOVA followed by Tukey’s post hoc test. A value of p<0.05 was considered significant.

## Results

### Cell culture

**Fig 3:**
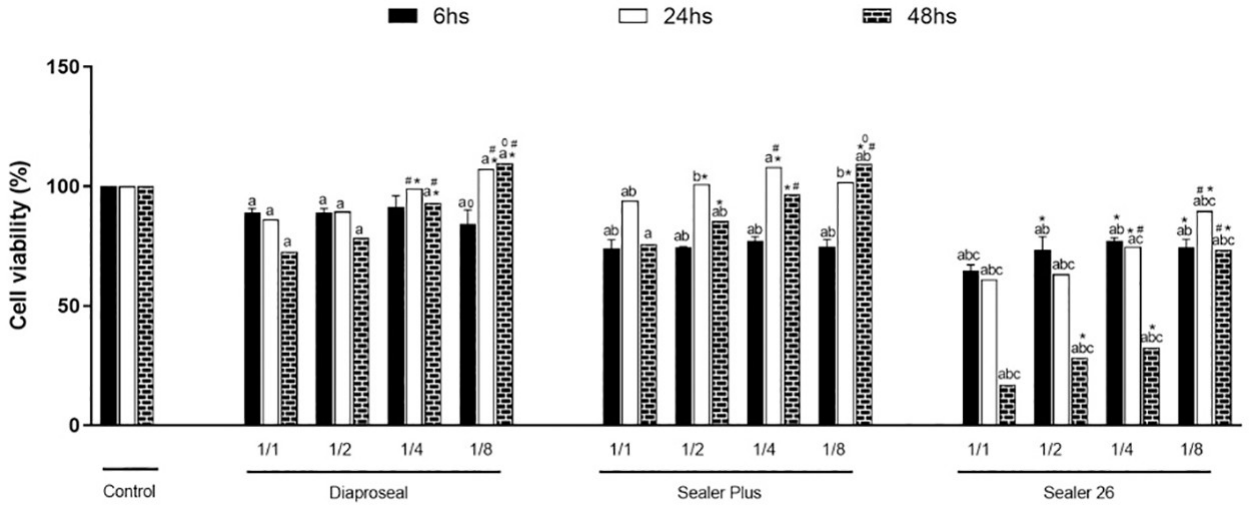
Cell proliferation after exposure to different dilution of sealers extract: The letters indicate statistical difference comparing different materials in the same dilution: a: vs. Control; b: vs. Dia-Proseal; c: vs. Sealer Plus. The symbols indicate statistical difference observed comparing different extract dilution of the same material: *: vs. undiluted extract; #: vs. ½ dilution; o: vs. ¼ dilution.

MTT assay revealed that cell proliferation was affected by sealers extract (undiluted, ½, ¼ and ⅛) in all time evaluated when compared with Control since a reduction on cell growth was observed (p<0.0001), except for Dia-Proseal ⅛ dilution, at 24h and 48h, and Sealer Plus ⅛ dilution that increased cell growth at 48h when compared with Control group (Fig 3).

Cell proliferation was dilution dependent since the cell exposure to higher dilution (¼ and ⅛) of sealers extract (Dia-Proseal, Sealer Plus) promoted a raise on cell metabolism at 24h and 48hs (p<0.0001). However, only at 6hs, Dia-Proseal ⅛ dilution promoted a decrease on cell growth when compared with Dia-Proseal ¼ dilution (p = 0.03) (Fig 3).

Comparison between sealers extracts at the same dilution showed that irrespective of Sealer 26 dilution (undiluted, ½, ¼ and ⅛) cell proliferation was negatively affected compared with Dia-Proseal and Sealer Plus extract once a decrease on cell growth was detected (p<0.0001). On the other hand, comparison among Dia-Proseal and Sealer Plus at the same dilution revealed that Dia-Proseal ¼ and ⅛ dilution stimulated cell growth at 6h (p<0.0001). Besides, a decrease, at 6h, and a raise at 24h and 48h (p = 0.03), on cell metabolism in presence of undiluted and ½ Sealer Plus was identified (Fig 3).

### Biocompatibility and biomineralization analysis

**Fig 4:**
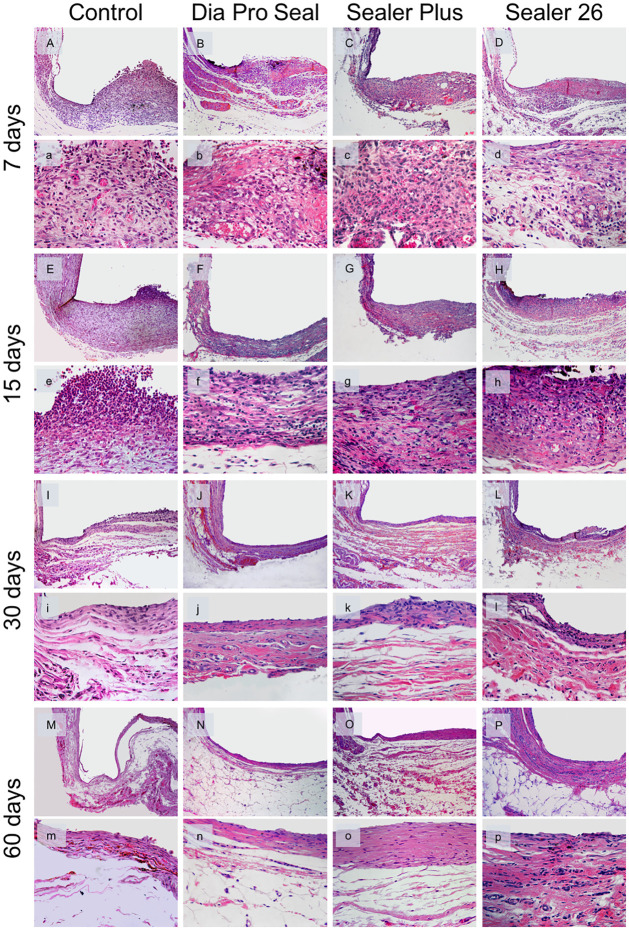
Representative images of subcutaneous inflammatory response. Samples were stained with HE and observed under an optical microscope (A–P ×100), (a–p ×400). The control group at 7 and 15 days: thick fibrous capsule and moderate inflammatory reaction (A,a/E,e); reduction in the thickness of the fibrous capsule and mild inflammatory reaction from day 30 (I,i) to 60 (M,m). Dia Pro Seal at 7 days: (B,b) thick fibrous capsule formation and moderate inflammatory cell infiltration; after 15 (F,f), 30 (J,j), and 60 (N,n) days: Reduction in the thickness of the fibrous capsule and mild inflammatory cell infiltration. Sealer Plus at 7 days (C,c) also shows thick fibrous capsule formation and moderate inflammation. After 15 days (G,g), a thin fibrous capsule was observed with moderate inflammatory reaction, which remained until day 30 (K,k). After 60 days (O,o) inflammatory infiltrate was mild in a thin fibrous capsule. Sealer 26 induced a thick fibrous capsule and a moderate inflammatory infiltrate at days 7 (D,d), 15 (H,h) and 30 (L,l), when inflammation started to decrease and fibrous capsule became thin, observed at day 60 (P,p).

Representative images of the tissue responses can be observed in Fig 4 and the data analysis in Table 2. At 7 days, the major specimens had moderate inflammatory infiltration, mainly polymorphonuclear and macrophage cells, in all groups. These cells were still present in the fibrous capsule, within 15 days. There was no significant difference between groups in these analysis periods (p>0.05). The fibrous capsule was thick in all groups at 7 days, but at 15 days, most of the specimens of Dia-ProSeal^®^ and Sealer Plus^®^ had thin fibrous capsules.

**Table 2:** Inflammation scores, thickness of the fibrous capsule and biomineralization (Von Kossa–VK / Polarized Light–PL) of the groups in each analysis periods.

Groups	Scores				Median*	P value	Fibrous capsule	Biomineralization (%)		*n*
	0	1	2	3				VK	PL	
**7 Days**										
Control	0	3	7	0	2^a^	= 0.311	Thick	0	0	10
Dia Pro Seal	0	4	6	0	2^a^		Thick	10	10	10
Sealer Plus	0	2	8	0	2^a^		Thick	10	10	10
Sealer 26	0	1	8	1	2^a^		Thick	100	100	10
**15 days**										
Control	0	4	6	0	2^a^	= 0.274	Thick	0	0	10
Dia Pro Seal	0	3	7	0	2^a^		Thin	10	10	10
Sealer Plus	0	5	5	0	1.5^a^		Thin	10	10	10
Sealer 26	0	1	9	0	2^a^		Thick	100	100	10
**30 days**										
Control	2	7	1	0	1^a^	= 0.006	Thin	0	0	10
Dia Pro Seal	3	4	3	0	1^a^		Thin	10	10	10
Sealer Plus	3	2	5	0	2^ab^		Thin	10	10	10
Sealer 26	1	4	5	0	2^b^		Thin	100	80	10
**60 Days**										
Control	4	6	0	0	1^a^	= 0.295	Thin	0	0	10
Dia Pro Seal	5	4	1	0	1^a^		Thin	10	0	10
Sealer Plus	6	4	0	0	0^a^		Thin	10	0	10
Sealer 26	2	7	1	0	1^a^		Thin	100	40	10

*Equal superscript letters (^a, b^) indicate no statistical difference while different superscript letters indicate significant difference between groups, regarding inflammation in each analysis period in each analysis period (*P* < 0.05).

At 30 days, the inflammatory infiltrate was mild in major specimens of the groups, except Sealer Plus and Sealer 26 groups, which had a moderate inflammatory infiltrate in this period. There is a significant difference between the Sealer 26 group, with greater inflammation, compared to Control and Dia-ProSeal^®^ groups (p = 0.006). At 60 days, groups showed no inflammation to mild inflammatory infiltrate in most specimens, with few lymphocytes and macrophages and no significant difference between groups (p>0.05). The fibrous capsule was thin at 30 and 60 days in all groups (Fig 4, Table 2).

**Fig 5:**
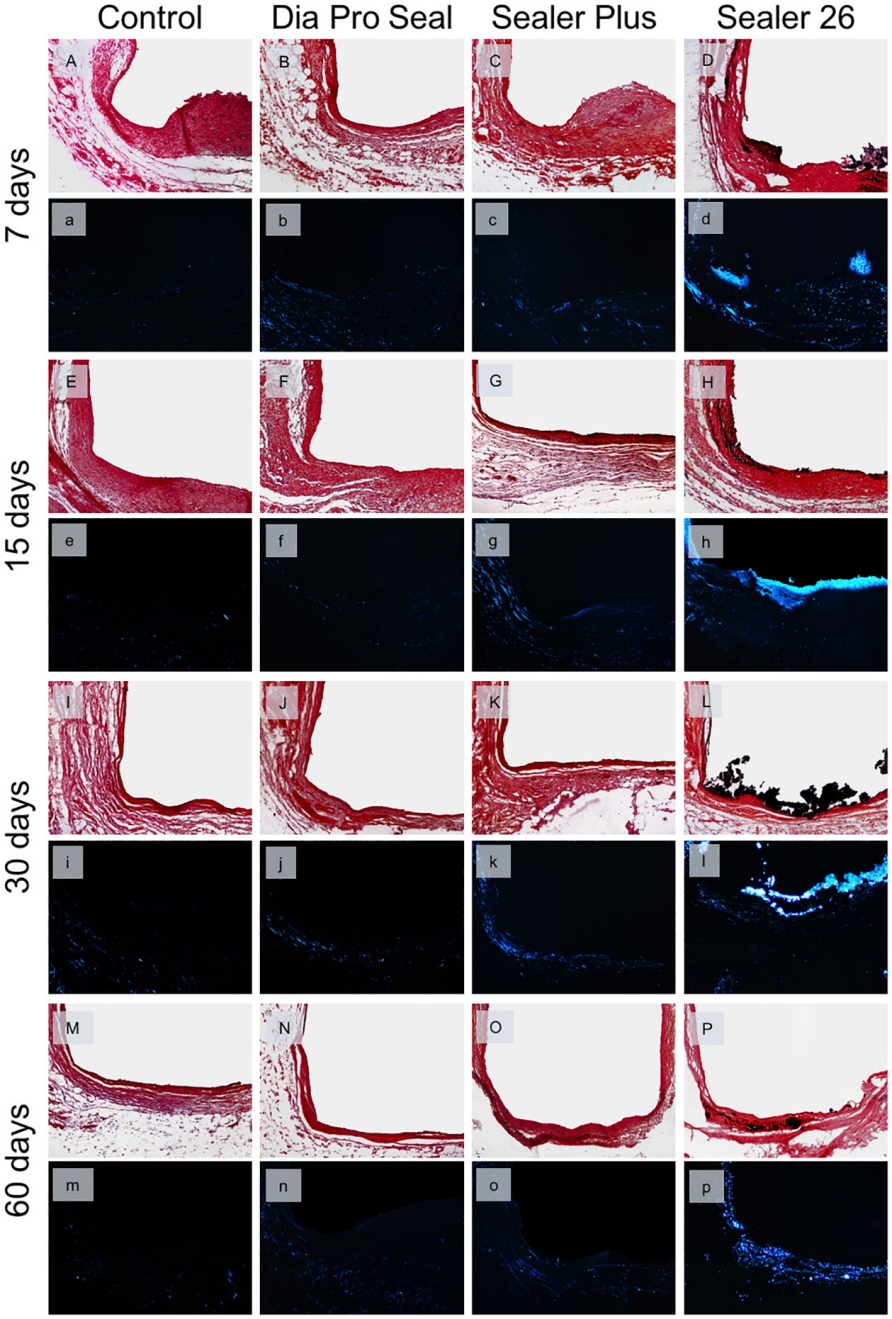
Representative images of biomineralization in Von Kossa (VK) staining and under polarized light (PL) (×100 magnification). Control at 7, 15, 30, and 60 days: Absence of dystrophic calcification (A,E,I,M) and absence of birefringent structures under polarized light near the tube opening (a,e,i,m). Dia Pro Seal group showed only a few samples with signs of mineralization after periods of 7 (B,b), 15 (F,f) and 30 (J,j) days for both mineralization analysis, reducing birefringent structures after 60 days (N,n). Similar results in Sealer Plus Group after 7 (C,c), 15 (G,c), 30 (K,k) and 60 (O,o) days. Sealer 26 showed Von Kossa positive staining and granulations birefringent to PL in all samples of days 7 (D,d) and 15 (H,h), with a reduction in birefringent structures at day 30 (L,l) and 60 (P,p).

Regarding the induction of mineralized tissue, only Sealer 26 was positive to Von Kossa staining in all samples (100%) through all experimental period, evidencing presence of biomineralization (Fig 5, Table 2). Also, the presence of birefringent structures under Polarized light was observed in 100% of samples only after 7 and 15 days, reducing to 80% from 30 days and 40% at the end of the experiment (Table 2). Although Sealer Plus and Dia ProSeal have calcium hydroxide in their composition, only 10% of analyzed samples from each sealer showed positive mineralized structures with VK staining through all experiment. Under Polarized light, the same 10% from each sealer was observed only after 7, 15 and 30 days. After 60 days, none of the analyzed samples from both sealers showed birefringence structures under PL (Table 2). The control group was negative in all samples for VK staining and birefringent structures in all experimental periods (Fig 5).

### Collagen maturation analysis

**Fig 6:**
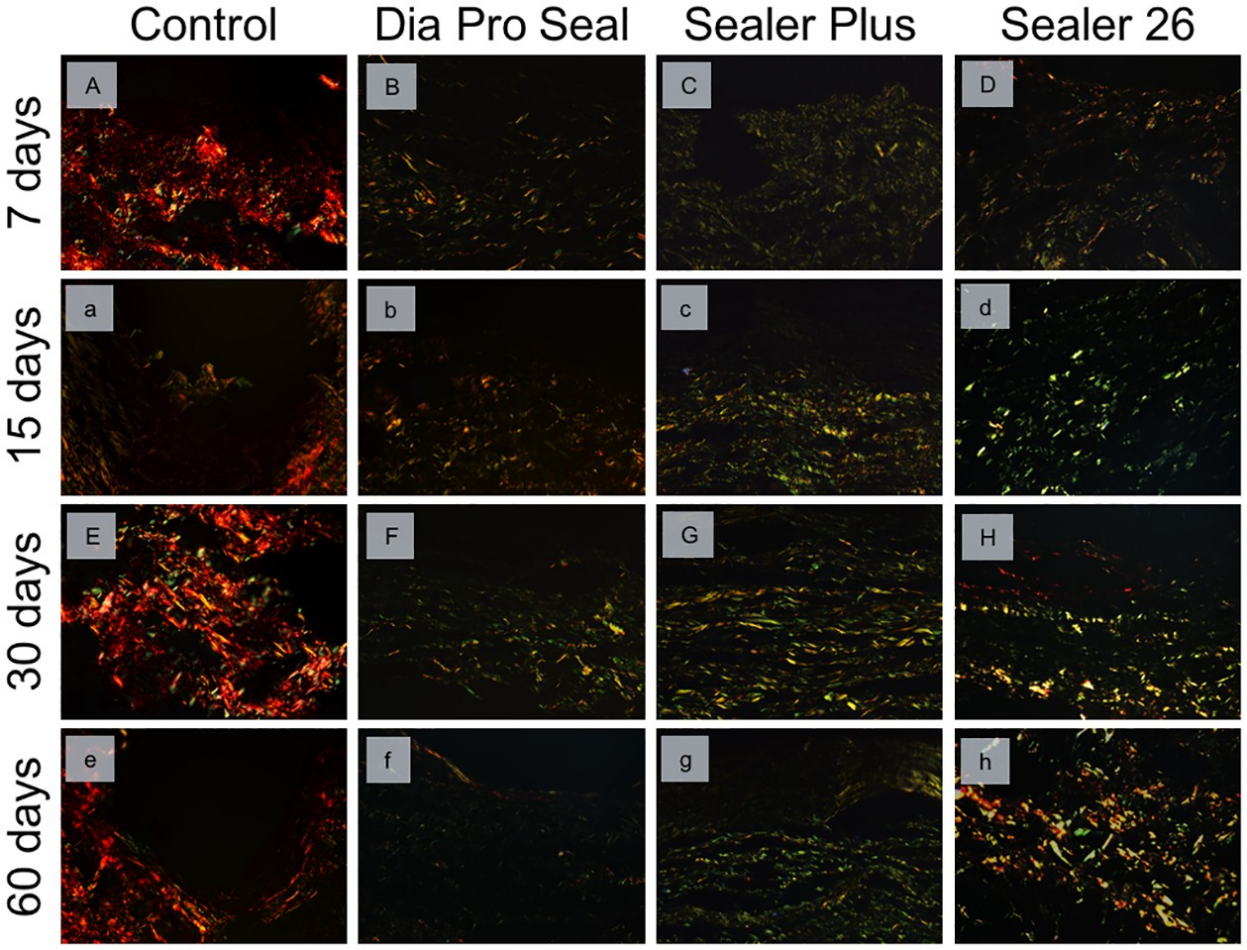
Representative images of mature and immature collagen fibers under Picrosirius Red staining (magnification ×400). At days 7 and 15, Dia Pro Seal (B,b), Sealer Plus (C,c) and Sealer 26 (D,d) showed significant presence of immature collagen fibers compared to the control group (A,a). At 30 and 60 days, the amount of mature fibers in the Sealer 26 (H,h) group increased, similar to the control group (E,e). After 60 days, the amount of mature fibers in Sealer 26 group (h) was superior compared to the Dia-ProSeal (f) and Sealer Plus (g) groups.

Representative images of the collagen fibers maturation can be observed in Fig 6 and the data analysis in Table 3. At 7 and 15 days, all experimental groups had a significant percentage of immature collagen fibers compared to the control group (p = 0.001). At 30 and 60 days, the amount of mature fibers in the Sealer 26 group increased, similar to the control group (p>0.05), especially at 60 days, where the amount of mature fibers this group was significant compared to the Dia-ProSeal^®^ and Sealer Plus^®^ groups (p<0.001).

**Table 3:** Percentage of mature and immature collagen fibers from each group at 7, 15, 30 and 60 days.

Groups	7 days			15 days			30 days			60 days		
	Mature	Immature	*	Mature	Immature	*	Mature	Immature	*	Mature	Immature	*
**Control**	70.95 ± 9.60	29.05 ± 9.60	^a^	49.48 ± 7.13	50.52 ± 7.13	^a^	78.89 ± 10.23	21.11 ± 10.23	^a^	75.00 ± 12.23	25.00 ± 12.23	^a^
**Dia Pro Seal**	23.99 ± 6.18	76.01 ± 6.18	^b^	22.24 ± 10.33	77.76 ± 10.33	^b^	22.64 ± 7.33	77.36 ± 7.33	^b^	20.83 ± 15.10	79.17 ± 15.10	^b^
**Sealer Plus**	15.93 ± 13.92	84.07 ± 13.92	^b^	30.95 ± 8.02	69.05 ± 8.02	^b^	26.73 ± 9.98	73.27 ± 9.98	^bc^	26.85 ± 7.91	73.15 ± 7.91	^b^
**Sealer 26**	26.47 ± 15.39	73.53 ± 15.39	^b^	32.48 ± 8.53	67.52 ± 8.53	^b^	52.94 ± 10.36	47.06 ± 10.36	^ac^	65.07 ± 6.08	34.93 ± 6.08	^a^
***P* value**	*P* = 0.001			*P* < 0.001			*P* < 0.001			*P* < 0.001		

*Equal superscript letters (^a, b, c^) in the columns indicate no statistical difference and different superscript letters indicate significant difference between groups, regarding maturation of collagen fibers in each analysis period (*P* < 0.05).

### Microbiological analysis

Table 4 presents the results of the microbiological analysis. The largest diameter of inhibition zone among the sealers, was observed in the Sealer 26^®^ group at 24 h and at 48 h. However, all experimental groups had a diameter of zone of inhibition significantly similar to that of the chlorhexidine group (positive control), with no significant difference between them (*P* = 1.000).

**Table 4:** Mean diameter of the inhibition zone (mm) in the groups at 24 and 48 hours.

Groups	24 h*	48 h*
**Chlorhexidine**	16.7 ± 1.16^a^	17.4 ± 1.50^a^
**Dia Pro Seal**	10.5 ± 0.88^a^	11.3 ± 0.77^a^
**Sealer Plus**	10.4 ± 0.42^a^	11.5 ± 1.21^a^
**Sealer 26**	15.2 ± 2.07^a^	16.5 ± 3.37^a^
***P* value**	*P* = 1.000	*P* = 1.000

*Same superscript letters (^a^) indicate no significant difference between groups in any period of analysis (*P* < 0.05).

## Discussion

This study investigated biological behavior of epoxy resin-based endodontic sealers containing calcium hydroxide. Based off results, since all sealers showed similar biological response, differing only in bioactivity potential, the null hypothesis was partially rejected.

Currently, several methodologies are used to assess properties of endodontic sealers. Both *in vitro* methodologies used in the present study were based upon previous reports assessing cytotoxicity [1,19,20] and antimicrobial potential against planktonic bacteria [21,22].

The cytotoxicity was evaluated by MTT assay, previously used to assess biocompatibility of endodontic sealers [23–26], in compliance with ISO 10993–5:2009 “Biological evaluation of medical devices. Part 5. Test for in vitro cytotoxicity”, which specify the incubation of cultured cells in directly contact with extracts.

Anterior findings using L929 fibroblast cell culture showed greater cell viability and less cytotoxicity for Sealer Plus than AH Plus, Simpli Seal and EndoFill [1], which is in accordance with our results: although undifferentiated pulp cells were used, cellular viability was higher in Sealer Plus and Dia-Proseal than Sealer 26 group. Combined with the initial HE results and biomineralization data, this cytotoxicity from Sealer 26 may be attribute to an elevated initial calcium ion release and consequently high pH. This assumption was supported by calcium ion release studies from Duarte et al. [27] and Tanomaru-Filho et al. [28], evidencing that Sealer 26 exhibits elevated Ca ions release during initial periods (setting time), leading to local alkalinity and cell injury by coagulative necrosis [29].

The methodology used to assess antimicrobial property was the agar diffusion test (ADT), which evaluates activity against planktonic bacteria (i.e. single cell isolates floating in specific medium), recognized as a basic test and used for several years [21,22,30–32]. AlShwaimi et al. [33] conducted a systematic review concerning *in vitro* antimicrobial effectiveness of root canal sealers and observed that, up to that date, most of studies concerning antimicrobial effect of endodontic sealers were performed on planktonic bacteria.

The chlorhexidine (CHX) was used as a positive control due to its antimicrobial activity [34,35]. In our study, although CHX showed the largest inhibition zone in both analyzed periods, followed by Sealer 26, no statistical difference was observed among groups. Our results regarding Sealer 26 antimicrobial activity are in accordance with previous findings with the same sealer, whereas in ADT or different methodologies [22,36,37]. Gomes et al. [38] reported a weaker antimicrobial activity of Sealer 26 with Direct Contact analysis 24 h after manipulation. However, authors compared with zinc oxide-eugenol (ZOE) based sealers, which has superior antibacterial activity, especially in this scenario where ZOE components can diffuse through agar [39].

However, it’s paramount to state that this methodology has several limitations, such as the diffusion dependency of tested materials. Also, the differentiation between zones of diffusion and inhibition may hinder analysis. This may be overcome with the used of triphenyltetrazolium, which indicates viable microorganisms (appear red in color), facilitating measurement precision [32,36]. In addition, planktonic bacteria may not simulate a clinical situation since, in oral environment, bacteria are organized in biofilm form, which provides protection during biofilm maturation and establishment in a hostile environment [22,33,40]. Still, it’s important to obtain initial data on antimicrobial activity of sealers but precipitate conclusions should not be drawn.

The *in vivo* methodology used to assess tissue response was the rat model. The use of polyethylene tubes implanted into subcutaneous connective tissue initiated with Torneck in the 60’s [41], posteriorly established as a standard methodology recommended by ISO 10993 [42]. Several studies reported the same *in vivo* model with polyethylene tubes to evaluate different tissue response such as inflammation and fibrous capsule [1,5,6,8,16,43], biomineralization [8,16,17,19] and collagen fiber maturation [17,44,45].

Regarding biocompatibility, the initial inflammatory reaction is partially resulted from the surgical trauma. A previous study showed satisfactory results after 30 days from Sealer Plus [1] compared to AH Plus and SimpliSeal. However, authors evaluated only after 7 and 30 days. In the present study, another two experimental periods of 15 and 60 days were observed. Although our results differed showing a moderate inflammatory infiltrate after 30 days from Sealer Plus, all sealers were biocompatible because initial inflammatory response decreased over time, corroborating the aforementioned research [1]. The same biocompatibility result of Sealer 26 was previously found after 42 days [46]. There is no *in vivo* biocompatibility data from Dia-ProSeal in literature, but the satisfactory outcome is related to its components, very similar to Sealer Plus.

With respect to thickness of fibrous capsule, samples of Sealer 26 showed a thick fibrous capsule until 30 days, while the other two sealers showed a thin capsule. According to results demonstrated by Santos et al. [47] with resinous and calcium silicate sealers, the fibrous capsule may be considered thick due to effects of mast cells on fibroblast proliferation and increase in macrophage infiltrate, observed in sealers with bioactive potential because tissue response to formation of calcific precipitates [48], which is in accordance with our results. Therefore, fibroblastic proliferation may be compatible with progressive resolution of the inflammatory reaction, as deposition of fibrous capsule around the material is an indicative of tissue tolerance [47].

The chemical reaction resulted from the dissociation of calcium hydroxide [Ca(OH)_2_] into calcium ions and hydroxyl allows an association with carbon dioxide (CO_2_) from living tissue, resulting in the formation of calcite crystals (calcium carbonate—CaCO_3_) in the surrounding area, elevating pH [5,16,17]. The induction of mineralized tissue deposition occurs when these calcite crystals trigger a biomineralization phenomenon with proteins of the extracellular matrix, which may stimulate bone defects closure by formation of calcified areas [17,19]. In addition, an antimicrobial property is attributed due to the use of calcium by the connective tissue, reducing the presence of local carbon dioxide, which is used by bacteria for anaerobic respiration [49].

The Von Kossa staining is used to observe formation of mineralized structures, darkly stained by this technique, while unstained specimens allow visualization of birefringent structures under polarized light, related to presence of calcium carbonate crystals. This methodology has been extensively used to assess biomineralization ability of endodontic materials such as MTA-like repair cements or endodontic obturating sealer [5,8,16,19,20].

In the present study, hard tissue deposition was observed mainly in Sealer 26 group through all experimental period. This result corroborates a previous research which evaluated presence of calcium salts deposition in the dorsa of rats with Sealer 26 and also reported mineralized structures under polarized light and Von Kossa analysis after 30 days [50]. Supporting the present methodology, Hinata et al. [51], analyzed with scanning electron microscopy-electron probe microanalysis (SEM-EPMA) the chemical compositions of the surface precipitates formed on subcutaneous implants with bioceramic cements, detecting calcium and phosphorus, and associating with the Von Kossa positive structures when biomineralization is observed after contact with connective tissue.

Interestingly, the biomineralization data also evidenced a reduction in birefringent structures under polarized light along the experimental period, especially in Sealer 26 group. Birefringent granulations were present in all samples (100%) of Sealer 26 at initial time periods, but decreased over time to 80% of samples after 30 days and 40% after 60 days. We attribute this fact to a probable reduction in calcium release after 30 days and on, remaining the initially mineralized structures observed by Von Kossa staining, which remained positive in all samples. This reduction of birefringent structures over time is also in accordance with the results reported by Bueno et al. [5] with a bioceramic endodontic sealer.

Although all sealers presented calcium hydroxide in their composition, this result is linked to the epoxy resin base. A previous report regarding resin-based sealer containing calcium hydroxide (Acroseal, Septodont, FR) also showed lack of mineralized tissue in all four experimental periods, due to the relative insolubility of the epoxy resin base in those sealers [5], evidencing the need for calcium hydroxide-based sealers to be minimally soluble to liberate hydroxyl and calcium ions [52]. Reinforcing these findings, the solubility of Sealer 26 was superior than epoxy resin-based sealer AH Plus [40], which also has two bisphenol epoxy resin in composition in both pastes, similar to Sealer Plus and Dia-ProSeal.

The similarity between Dia-ProSeal and Sealer Plus composition with resin bisphenol A and F clarifies the low biomineralization results observed in this study for both sealers, also explained by the amount of calcium hydroxide reported in those sealers composition, as Sealer 26 shows 37%, while Sealer Plus and Dia ProSeal around 15% (Table 1).

Simultaneously, analysis of collagen maturation was performed using polarized light microscopy. The PSR technique enhances the natural birefringence of collagen fibers, since collagen molecules rich in basic amino acids react strongly with acid dyes such as Sirius Red, differing collagen types by color [53], as greenish-yellow suggests that collagen is immature and thin while a yellowish-red color suggests better maturity and thick fiber organization [17,44,45].

In sealer 26 group, an increase in mature collagen fiber was clearly observed from day 7 to 60, indicating dense connective tissue formation over time, which associated with reduction of inflammatory infiltrate may induce a response that culminates in tissue repair. In this group, the increase of mature fiber after days 30 and 60 may be correlated with the probable reduction in calcium release after 30 days, observed by reduction of birefringent structures under PL.

Lastly, it’s also important to highlight the limitations of the present research as *in vitro* and *in vivo* animal models are considered as preliminary studies for new materials. Further studies with physico-chemical properties such as solubility, radiopacity, pH measurement, setting time and more accurate biomineralization and inflammatory investigation with immunomarkers are highly indicated. Also, additional antimicrobial research with more complex biofilm models is necessary to complement the present findings.

## Conclusion

Within the limitation of this study, all sealers showed *in vitro* and *in vivo* biocompatibility. The sealer 26 induced more biomineralization than Sealer Plus and Dia ProSeal. All sealers showed antimicrobial activity against planktonic bacteria. In this animal model, when deposition of mineralized tissue is required, Sealer 26 showed most favorable indication.

## Supporting information

S1 File Cell culture. Statistical results provided by SigmaPlot statistical software with significance.
(PDF)

S2 File Antimicrobial activity. Inhibitory halo induced by groups, measured by digital caliper.
(PDF)

S3 File Inflammatory response. Inflammatory score (1–3) induced by collected samples.
(PDF)

S4 File Collagen repair. Red and Green fiber area in each group and final fiber percentage.
(PDF)
