# Supplementary material for: Biological investigation of resinous endodontic sealers containing calcium hydroxide
Source: PLoS One. 2023 Jul 17;18(7):e0287890. doi: 10.1371/journal.pone.0287890 (PMC10351732; doi:10.1371/journal.pone.0287890)
Supplement: S1 File — Statistical results provided by SigmaPlot statistical software with significance. (PDF) [file pone.0287890.s001.pdf]

Parameter	
Table Analyzed	Data 1

Two-way ANOVA		Ordinary
Alpha	0,05	

Source of Variation		% of total variation		P value	P value summary	Significant?
Interaction	26,87	<0,0001	****	Yes		
Row Factor	9,129	<0,0001	****	Yes		
Column Factor		62,79	<0,0001	****	Yes	

ANOVA table	SS (Type III)	DF	MS	F (DFn, DFd)	P value	
Interaction	11889	24	495,4	F (24, 61) = 57,07	P<0,0001	
Row Factor	4039	2	2020	F (2, 61) = 232,7	P<0,0001	
Column Factor		27782	12	2315	F (12, 61) = 266,7	P<0,0001
Residual	529,5	61	8,680			

Data summary	
Number of columns (Column Factor)	13
Number of rows (Row Factor)	3
Number of values	100

Within each row, compare columns (simple effects within rows)

Number of families 1
Number of comparisons per family 234
Alpha 0,05

Bonferroni's multiple comparisons test Predicted (LS) mean diff, 95,00% CI of diff, Below threshold? Summary

Bonferroni's multiple comparisons test	Predicted (LS) mean diff,	95,00% CI of diff,	Below threshold?	Summary
6hs				
Diaproseal 100 vs. Control	-11,04	-21,63 to -0,4440	Yes	*
Diaproseal 1/2 vs. Control	-11,04	-21,63 to -0,4440	Yes	*
Diaproseal 1/4 vs. Control	-5,893	-15,37 to 3,580	No	ns
Diaproseal 1/8 vs. Control	-15,63	-25,11 to -6,160	Yes	****
Sealer Plus 100 vs. Control	-26,00	-36,59 to -15,41	Yes	****
Sealer Plus 1/2 vs. Control	-25,53	-36,12 to -14,94	Yes	****
Sealer Plus 1/4 vs. Control	-22,82	-33,41 to -12,23	Yes	****
Sealer Plus 1/8 vs. Control	-25,22	-34,69 to -15,75	Yes	****
Sealer 26 100 vs. Control	-35,19	-44,66 to -25,72	Yes	****
Sealer 26 1/2 vs. Control	-26,55	-36,03 to -17,08	Yes	****
Sealer 26 1/4 vs. Control	-22,86	-32,33 to -13,39	Yes	****
Sealer 26 1/8 vs. Control	-25,29	-34,77 to -15,82	Yes	****
Diaproseal 1/2 vs. Diaproseal 100	0,000	-11,60 to 11,60	No	ns
Diaproseal 1/4 vs. Diaproseal 100	5,142	-5,449 to 15,73	No	ns
Diaproseal 1/8 vs. Diaproseal 100	-4,598	-15,19 to 5,993	No	ns
Sealer Plus 100 vs. Diaproseal 100	-14,97	-26,57 to -3,363	Yes	***
Sealer Plus 1/2 vs. Diaproseal 100	-14,50	-26,10 to -2,893	Yes	**

Sealer Plus 1/4 vs. Diaproseal 100	-11,79	-23,39 to -0,1832	Yes	*
Sealer Plus 1/8 vs. Diaproseal 100	-14,19	-24,78 to -3,594	Yes	***
Sealer 26 100 vs. Diaproseal 100	-24,16	-34,75 to -13,56	Yes	****
Sealer 26 1/2 vs. Diaproseal 100	-15,52	-26,11 to -4,927	Yes	****
Sealer 26 1/4 vs. Diaproseal 100	-11,83	-22,42 to -1,234	Yes	*
Sealer 26 1/8 vs. Diaproseal 100	-14,26	-24,85 to -3,667	Yes	***
Diaproseal 1/4 vs. Diaproseal 1/2	5,142	-5,449 to 15,73	No	ns
Diaproseal 1/8 vs. Diaproseal 1/2	-4,598	-15,19 to 5,993	No	ns
Sealer Plus 100 vs. Diaproseal 1/2	-14,97	-26,57 to -3,363	Yes	***
Sealer Plus 1/2 vs. Diaproseal 1/2	-14,50	-26,10 to -2,893	Yes	**
Sealer Plus 1/4 vs. Diaproseal 1/2	-11,79	-23,39 to -0,1832	Yes	*
Sealer Plus 1/8 vs. Diaproseal 1/2	-14,19	-24,78 to -3,594	Yes	***
Sealer 26 100 vs. Diaproseal 1/2	-24,16	-34,75 to -13,56	Yes	****
Sealer 26 1/2 vs. Diaproseal 1/2	-15,52	-26,11 to -4,927	Yes	****
Sealer 26 1/4 vs. Diaproseal 1/2	-11,83	-22,42 to -1,234	Yes	*
Sealer 26 1/8 vs. Diaproseal 1/2	-14,26	-24,85 to -3,667	Yes	***
Diaproseal 1/8 vs. Diaproseal 1/4	-9,740	-19,21 to -0,2672	Yes	*
Sealer Plus 100 vs. Diaproseal 1/4	-20,11	-30,70 to -9,516	Yes	****
Sealer Plus 1/2 vs. Diaproseal 1/4	-19,64	-30,23 to -9,046	Yes	****
Sealer Plus 1/4 vs. Diaproseal 1/4	-16,93	-27,52 to -6,336	Yes	****
Sealer Plus 1/8 vs. Diaproseal 1/4	-19,33	-28,80 to -9,854	Yes	****
Sealer 26 100 vs. Diaproseal 1/4	-29,30	-38,77 to -19,82	Yes	****
Sealer 26 1/2 vs. Diaproseal 1/4	-20,66	-30,13 to -11,19	Yes	****

Sealer 26 1/4 vs. Diaproseal 1/4	-16,97	-26,44 to -7,494	Yes	****
Sealer 26 1/8 vs. Diaproseal 1/4	-19,40	-28,87 to -9,927	Yes	****
Sealer Plus 100 vs. Diaproseal 1/8	-10,37	-20,96 to 0,2243	No	ns
Sealer Plus 1/2 vs. Diaproseal 1/8	-9,897	-20,49 to 0,6943	No	ns
Sealer Plus 1/4 vs. Diaproseal 1/8	-7,187	-17,78 to 3,404	No	ns
Sealer Plus 1/8 vs. Diaproseal 1/8	-9,587	-19,06 to -0,1138	Yes	*
Sealer 26 100 vs. Diaproseal 1/8	-19,56	-29,03 to -10,08	Yes	****
Sealer 26 1/2 vs. Diaproseal 1/8	-10,92	-20,39 to -1,447	Yes	**
Sealer 26 1/4 vs. Diaproseal 1/8	-7,227	-16,70 to 2,246	No	ns
Sealer 26 1/8 vs. Diaproseal 1/8	-9,660	-19,13 to -0,1872	Yes	*
Sealer Plus 1/2 vs. Sealer Plus 100	0,4700	-11,13 to 12,07	No	ns
Sealer Plus 1/4 vs. Sealer Plus 100	3,180	-8,422 to 14,78	No	ns
Sealer Plus 1/8 vs. Sealer Plus 100	0,7800	-9,811 to 11,37	No	ns
Sealer 26 100 vs. Sealer Plus 100	-9,190	-19,78 to 1,401	No	ns
Sealer 26 1/2 vs. Sealer Plus 100	-0,5533	-11,14 to 10,04	No	ns
Sealer 26 1/4 vs. Sealer Plus 100	3,140	-7,451 to 13,73	No	ns
Sealer 26 1/8 vs. Sealer Plus 100	0,7067	-9,884 to 11,30	No	ns
Sealer Plus 1/4 vs. Sealer Plus 1/2	2,710	-8,892 to 14,31	No	ns
Sealer Plus 1/8 vs. Sealer Plus 1/2	0,3100	-10,28 to 10,90	No	ns
Sealer 26 100 vs. Sealer Plus 1/2	-9,660	-20,25 to 0,9310	No	ns
Sealer 26 1/2 vs. Sealer Plus 1/2	-1,023	-11,61 to 9,568	No	ns
Sealer 26 1/4 vs. Sealer Plus 1/2	2,670	-7,921 to 13,26	No	ns
Sealer 26 1/8 vs. Sealer Plus 1/2	0,2367	-10,35 to 10,83	No	ns

Sealer Plus 1/8 vs. Sealer Plus 1/4	-2,400	-12,99 to 8,191	No	ns
Sealer 26 100 vs. Sealer Plus 1/4	-12,37	-22,96 to -1,779	Yes	**
Sealer 26 1/2 vs. Sealer Plus 1/4	-3,733	-14,32 to 6,858	No	ns
Sealer 26 1/4 vs. Sealer Plus 1/4	-0,04000	-10,63 to 10,55	No	ns
Sealer 26 1/8 vs. Sealer Plus 1/4	-2,473	-13,06 to 8,118	No	ns
Sealer 26 100 vs. Sealer Plus 1/8	-9,970	-19,44 to -0,4972	Yes	*
Sealer 26 1/2 vs. Sealer Plus 1/8	-1,333	-10,81 to 8,140	No	ns
Sealer 26 1/4 vs. Sealer Plus 1/8	2,360	-7,113 to 11,83	No	ns
Sealer 26 1/8 vs. Sealer Plus 1/8	-0,07333	-9,546 to 9,400	No	ns
Sealer 26 1/2 vs. Sealer 26 100	8,637	-0,8362 to 18,11	No	ns
Sealer 26 1/4 vs. Sealer 26 100	12,33	2,857 to 21,80	Yes	***
Sealer 26 1/8 vs. Sealer 26 100	9,897	0,4238 to 19,37	Yes	*
Sealer 26 1/4 vs. Sealer 26 1/2	3,693	-5,780 to 13,17	No	ns
Sealer 26 1/8 vs. Sealer 26 1/2	1,260	-8,213 to 10,73	No	ns
Sealer 26 1/8 vs. Sealer 26 1/4	-2,433	-11,91 to 7,040	No	ns

24hs

Diaproseal 100 vs. Control	-13,85	-23,32 to -4,377	Yes	****
Diaproseal 1/2 vs. Control	-10,56	-20,04 to -1,090	Yes	*
Diaproseal 1/4 vs. Control	2,350	-8,241 to 12,94	No	ns
Diaproseal 1/8 vs. Control	7,390	-3,201 to 17,98	No	ns
Sealer Plus 100 vs. Control	-6,023	-15,50 to 3,450	No	ns
Sealer Plus 1/2 vs. Control	1,097	-8,376 to 10,57	No	ns
Sealer Plus 1/4 vs. Control	8,100	-2,491 to 18,69	No	ns

Sealer Plus 1/8 vs. Control	1,875	-8,716 to 12,47	No	ns
Sealer 26 100 vs. Control	-38,97	-49,56 to -28,38	Yes	****
Sealer 26 1/2 vs. Control	-36,70	-46,17 to -27,22	Yes	****
Sealer 26 1/4 vs. Control	-25,20	-34,67 to -15,72	Yes	****
Sealer 26 1/8 vs. Control	-10,21	-20,80 to 0,3810	No	ns
Diaproseal 1/2 vs. Diaproseal 100	3,287	-6,186 to 12,76	No	ns
Diaproseal 1/4 vs. Diaproseal 100	16,20	5,609 to 26,79	Yes	****
Diaproseal 1/8 vs. Diaproseal 100	21,24	10,65 to 31,83	Yes	****
Sealer Plus 100 vs. Diaproseal 100	7,827	-1,646 to 17,30	No	ns
Sealer Plus 1/2 vs. Diaproseal 100	14,95	5,474 to 24,42	Yes	****
Sealer Plus 1/4 vs. Diaproseal 100	21,95	11,36 to 32,54	Yes	****
Sealer Plus 1/8 vs. Diaproseal 100	15,73	5,134 to 26,32	Yes	****
Sealer 26 100 vs. Diaproseal 100	-25,12	-35,71 to -14,53	Yes	****
Sealer 26 1/2 vs. Diaproseal 100	-22,85	-32,32 to -13,37	Yes	****
Sealer 26 1/4 vs. Diaproseal 100	-11,35	-20,82 to -1,874	Yes	**
Sealer 26 1/8 vs. Diaproseal 100	3,640	-6,951 to 14,23	No	ns
Diaproseal 1/4 vs. Diaproseal 1/2	12,91	2,322 to 23,50	Yes	**
Diaproseal 1/8 vs. Diaproseal 1/2	17,95	7,362 to 28,54	Yes	****
Sealer Plus 100 vs. Diaproseal 1/2	4,540	-4,933 to 14,01	No	ns
Sealer Plus 1/2 vs. Diaproseal 1/2	11,66	2,187 to 21,13	Yes	**
Sealer Plus 1/4 vs. Diaproseal 1/2	18,66	8,072 to 29,25	Yes	****
Sealer Plus 1/8 vs. Diaproseal 1/2	12,44	1,847 to 23,03	Yes	**
Sealer 26 100 vs. Diaproseal 1/2	-28,41	-39,00 to -17,82	Yes	****

Sealer 26 1/2 vs. Diaproseal 1/2	-26,13	-35,61 to -16,66	Yes	****
Sealer 26 1/4 vs. Diaproseal 1/2	-14,63	-24,11 to -5,160	Yes	****
Sealer 26 1/8 vs. Diaproseal 1/2	0,3533	-10,24 to 10,94	No	ns
Diaproseal 1/8 vs. Diaproseal 1/4	5,040	-6,562 to 16,64	No	ns
Sealer Plus 100 vs. Diaproseal 1/4	-8,373	-18,96 to 2,218	No	ns
Sealer Plus 1/2 vs. Diaproseal 1/4	-1,253	-11,84 to 9,338	No	ns
Sealer Plus 1/4 vs. Diaproseal 1/4	5,750	-5,852 to 17,35	No	ns
Sealer Plus 1/8 vs. Diaproseal 1/4	-0,4750	-12,08 to 11,13	No	ns
Sealer 26 100 vs. Diaproseal 1/4	-41,32	-52,92 to -29,72	Yes	****
Sealer 26 1/2 vs. Diaproseal 1/4	-39,05	-49,64 to -28,46	Yes	****
Sealer 26 1/4 vs. Diaproseal 1/4	-27,55	-38,14 to -16,96	Yes	****
Sealer 26 1/8 vs. Diaproseal 1/4	-12,56	-24,16 to -0,9582	Yes	*
Sealer Plus 100 vs. Diaproseal 1/8	-13,41	-24,00 to -2,822	Yes	**
Sealer Plus 1/2 vs. Diaproseal 1/8	-6,293	-16,88 to 4,298	No	ns
Sealer Plus 1/4 vs. Diaproseal 1/8	0,7100	-10,89 to 12,31	No	ns
Sealer Plus 1/8 vs. Diaproseal 1/8	-5,515	-17,12 to 6,087	No	ns
Sealer 26 100 vs. Diaproseal 1/8	-46,36	-57,96 to -34,76	Yes	****
Sealer 26 1/2 vs. Diaproseal 1/8	-44,09	-54,68 to -33,50	Yes	****
Sealer 26 1/4 vs. Diaproseal 1/8	-32,59	-43,18 to -22,00	Yes	****
Sealer 26 1/8 vs. Diaproseal 1/8	-17,60	-29,20 to -5,998	Yes	****
Sealer Plus 1/2 vs. Sealer Plus 100	7,120	-2,353 to 16,59	No	ns
Sealer Plus 1/4 vs. Sealer Plus 100	14,12	3,532 to 24,71	Yes	***
Sealer Plus 1/8 vs. Sealer Plus 100	7,898	-2,693 to 18,49	No	ns

Sealer 26 100 vs. Sealer Plus 100	-32,95	-43,54 to -22,36	Yes	****
Sealer 26 1/2 vs. Sealer Plus 100	-30,67	-40,15 to -21,20	Yes	****
Sealer 26 1/4 vs. Sealer Plus 100	-19,17	-28,65 to -9,700	Yes	****
Sealer 26 1/8 vs. Sealer Plus 100	-4,187	-14,78 to 6,404	No	ns
Sealer Plus 1/4 vs. Sealer Plus 1/2	7,003	-3,588 to 17,59	No	ns
Sealer Plus 1/8 vs. Sealer Plus 1/2	0,7783	-9,813 to 11,37	No	ns
Sealer 26 100 vs. Sealer Plus 1/2	-40,07	-50,66 to -29,48	Yes	****
Sealer 26 1/2 vs. Sealer Plus 1/2	-37,79	-47,27 to -28,32	Yes	****
Sealer 26 1/4 vs. Sealer Plus 1/2	-26,29	-35,77 to -16,82	Yes	****
Sealer 26 1/8 vs. Sealer Plus 1/2	-11,31	-21,90 to -0,7157	Yes	*
Sealer Plus 1/8 vs. Sealer Plus 1/4	-6,225	-17,83 to 5,377	No	ns
Sealer 26 100 vs. Sealer Plus 1/4	-47,07	-58,67 to -35,47	Yes	****
Sealer 26 1/2 vs. Sealer Plus 1/4	-44,80	-55,39 to -34,21	Yes	****
Sealer 26 1/4 vs. Sealer Plus 1/4	-33,30	-43,89 to -22,71	Yes	****
Sealer 26 1/8 vs. Sealer Plus 1/4	-18,31	-29,91 to -6,708	Yes	****
Sealer 26 100 vs. Sealer Plus 1/8	-40,85	-52,45 to -29,24	Yes	****
Sealer 26 1/2 vs. Sealer Plus 1/8	-38,57	-49,16 to -27,98	Yes	****
Sealer 26 1/4 vs. Sealer Plus 1/8	-27,07	-37,66 to -16,48	Yes	****
Sealer 26 1/8 vs. Sealer Plus 1/8	-12,09	-23,69 to -0,4832	Yes	*
Sealer 26 1/2 vs. Sealer 26 100	2,273	-8,318 to 12,86	No	ns
Sealer 26 1/4 vs. Sealer 26 100	13,77	3,182 to 24,36Yes	***	
Sealer 26 1/8 vs. Sealer 26 100	28,76	17,16 to 40,36Yes	****	
Sealer 26 1/4 vs. Sealer 26 1/2	11,50	2,027 to 20,97Yes	**	

Sealer 26 1/8 vs. Sealer 26 1/2	26,49	15,90 to 37,08	Yes	****
Sealer 26 1/8 vs. Sealer 26 1/4	14,99	4,396 to 25,58	Yes	***

48hs

Diaproseal 100 vs. Control	-27,18	-36,65 to -17,70	Yes	****
Diaproseal 1/2 vs. Control	-21,49	-30,96 to -12,01	Yes	****
Diaproseal 1/4 vs. Control	-6,815	-17,41 to 3,776	No	ns
Diaproseal 1/8 vs. Control	9,745	-0,8460 to 20,34	No	ns
Sealer Plus 100 vs. Control	-24,12	-33,59 to -14,64	Yes	****
Sealer Plus 1/2 vs. Control	-14,52	-24,00 to -5,050	Yes	****
Sealer Plus 1/4 vs. Control	-3,375	-13,97 to 7,216	No	ns
Sealer Plus 1/8 vs. Control	9,490	-1,101 to 20,08	No	ns
Sealer 26 100 vs. Control	-83,10	-92,57 to -73,63	Yes	****
Sealer 26 1/2 vs. Control	-71,76	-81,23 to -62,29	Yes	****
Sealer 26 1/4 vs. Control	-65,61	-76,20 to -55,01	Yes	****
Sealer 26 1/8 vs. Control	-26,56	-37,15 to -15,97	Yes	****
Diaproseal 1/2 vs. Diaproseal 100	5,690	-3,783 to 15,16	No	ns
Diaproseal 1/4 vs. Diaproseal 100	20,36	9,771 to 30,95	Yes	****
Diaproseal 1/8 vs. Diaproseal 100	36,92	26,33 to 47,51	Yes	****
Sealer Plus 100 vs. Diaproseal 100	3,060	-6,413 to 12,53	No	ns
Sealer Plus 1/2 vs. Diaproseal 100	12,65	3,180 to 22,13	Yes	***
Sealer Plus 1/4 vs. Diaproseal 100	23,80	13,21 to 34,39	Yes	****
Sealer Plus 1/8 vs. Diaproseal 100	36,67	26,08 to 47,26	Yes	****
Sealer 26 100 vs. Diaproseal 100	-55,92	-65,40 to -46,45	Yes	****

Sealer 26 1/2 vs. Diaproseal 100	-44,58	-54,06 to -35,11	Yes	****
Sealer 26 1/4 vs. Diaproseal 100	-38,43	-49,02 to -27,84	Yes	****
Sealer 26 1/8 vs. Diaproseal 100	0,6167	-9,974 to 11,21	No	ns
Diaproseal 1/4 vs. Diaproseal 1/2	14,67	4,081 to 25,26Yes	***	
Diaproseal 1/8 vs. Diaproseal 1/2	31,23	20,64 to 41,82Yes	****	
Sealer Plus 100 vs. Diaproseal 1/2	-2,630	-12,10 to 6,843	No	ns
Sealer Plus 1/2 vs. Diaproseal 1/2	6,963	-2,510 to 16,44	No	ns
Sealer Plus 1/4 vs. Diaproseal 1/2	18,11	7,521 to 28,70Yes	****	
Sealer Plus 1/8 vs. Diaproseal 1/2	30,98	20,39 to 41,57Yes	****	
Sealer 26 100 vs. Diaproseal 1/2	-61,61	-71,09 to -52,14	Yes	****
Sealer 26 1/2 vs. Diaproseal 1/2	-50,27	-59,75 to -40,80	Yes	****
Sealer 26 1/4 vs. Diaproseal 1/2	-44,12	-54,71 to -33,53	Yes	****
Sealer 26 1/8 vs. Diaproseal 1/2	-5,073	-15,66 to 5,518	No	ns
Diaproseal 1/8 vs. Diaproseal 1/4	16,56	4,958 to 28,16Yes	***	
Sealer Plus 100 vs. Diaproseal 1/4	-17,30	-27,89 to -6,711	Yes	****
Sealer Plus 1/2 vs. Diaproseal 1/4	-7,708	-18,30 to 2,883	No	ns
Sealer Plus 1/4 vs. Diaproseal 1/4	3,440	-8,162 to 15,04	No	ns
Sealer Plus 1/8 vs. Diaproseal 1/4	16,31	4,703 to 27,91Yes	***	
Sealer 26 100 vs. Diaproseal 1/4	-76,29	-86,88 to -65,69	Yes	****
Sealer 26 1/2 vs. Diaproseal 1/4	-64,95	-75,54 to -54,35	Yes	****
Sealer 26 1/4 vs. Diaproseal 1/4	-58,79	-70,39 to -47,19	Yes	****
Sealer 26 1/8 vs. Diaproseal 1/4	-19,75	-31,35 to -8,143	Yes	****
Sealer Plus 100 vs. Diaproseal 1/8	-33,86	-44,45 to -23,27	Yes	****

Sealer Plus 1/2 vs. Diaproseal 1/8	-24,27	-34,86 to -13,68	Yes	****
Sealer Plus 1/4 vs. Diaproseal 1/8	-13,12	-24,72 to -1,518	Yes	**
Sealer Plus 1/8 vs. Diaproseal 1/8	-0,2550	-11,86 to 11,35	No	ns
Sealer 26 100 vs. Diaproseal 1/8	-92,85	-103,4 to -82,25	Yes	****
Sealer 26 1/2 vs. Diaproseal 1/8	-81,51	-92,10 to -70,91	Yes	****
Sealer 26 1/4 vs. Diaproseal 1/8	-75,35	-86,95 to -63,75	Yes	****
Sealer 26 1/8 vs. Diaproseal 1/8	-36,31	-47,91 to -24,70	Yes	****
Sealer Plus 1/2 vs. Sealer Plus 100	9,593	0,1205 to 19,07	Yes	*
Sealer Plus 1/4 vs. Sealer Plus 100	20,74	10,15 to 31,33	Yes	****
Sealer Plus 1/8 vs. Sealer Plus 100	33,61	23,02 to 44,20	Yes	****
Sealer 26 100 vs. Sealer Plus 100	-58,98	-68,46 to -49,51	Yes	****
Sealer 26 1/2 vs. Sealer Plus 100	-47,64	-57,12 to -38,17	Yes	****
Sealer 26 1/4 vs. Sealer Plus 100	-41,49	-52,08 to -30,90	Yes	****
Sealer 26 1/8 vs. Sealer Plus 100	-2,443	-13,03 to 8,148	No	ns
Sealer Plus 1/4 vs. Sealer Plus 1/2	11,15	0,5574 to 21,74	Yes	*
Sealer Plus 1/8 vs. Sealer Plus 1/2	24,01	13,42 to 34,60	Yes	****
Sealer 26 100 vs. Sealer Plus 1/2	-68,58	-78,05 to -59,10	Yes	****
Sealer 26 1/2 vs. Sealer Plus 1/2	-57,24	-66,71 to -47,76	Yes	****
Sealer 26 1/4 vs. Sealer Plus 1/2	-51,08	-61,67 to -40,49	Yes	****
Sealer 26 1/8 vs. Sealer Plus 1/2	-12,04	-22,63 to -1,446	Yes	**
Sealer Plus 1/8 vs. Sealer Plus 1/4	12,87	1,263 to 24,47	Yes	*
Sealer 26 100 vs. Sealer Plus 1/4	-79,73	-90,32 to -69,13	Yes	****
Sealer 26 1/2 vs. Sealer Plus 1/4	-68,39	-78,98 to -57,79	Yes	****

Sealer 26 1/4 vs. Sealer Plus 1/4	-62,23	-73,83 to -50,63	Yes	****
Sealer 26 1/8 vs. Sealer Plus 1/4	-23,19	-34,79 to -11,58	Yes	****
Sealer 26 100 vs. Sealer Plus 1/8	-92,59	-103,2 to -82,00	Yes	****
Sealer 26 1/2 vs. Sealer Plus 1/8	-81,25	-91,84 to -70,66	Yes	****
Sealer 26 1/4 vs. Sealer Plus 1/8	-75,10	-86,70 to -63,49	Yes	****
Sealer 26 1/8 vs. Sealer Plus 1/8	-36,05	-47,65 to -24,45	Yes	****
Sealer 26 1/2 vs. Sealer 26 100	11,34	1,867 to 20,81	Yes	**
Sealer 26 1/4 vs. Sealer 26 100	17,50	6,904 to 28,09	Yes	****
Sealer 26 1/8 vs. Sealer 26 100	56,54	45,95 to 67,13	Yes	****
Sealer 26 1/4 vs. Sealer 26 1/2	6,155	-4,436 to 16,75	No	ns
Sealer 26 1/8 vs. Sealer 26 1/2	45,20	34,61 to 55,79	Yes	****
Sealer 26 1/8 vs. Sealer 26 1/4	39,05	27,44 to 50,65	Yes	****

Test details	Predicted (LS) mean 1	Predicted (LS) mean 2	Predicted (LS) mean diff,	SE of diff,	N1	N2	t	DF
6hs								
Diaproseal 100 vs. Control	88,97	100,0	-11,04	2,689	2	3	4,103	61,00
Diaproseal 1/2 vs. Control	88,97	100,0	-11,04	2,689	2	3	4,103	61,00
Diaproseal 1/4 vs. Control	94,11	100,0	-5,893	2,406	3	3	2,450	61,00
Diaproseal 1/8 vs. Control	84,37	100,0	-15,63	2,406	3	3	6,499	61,00
Sealer Plus 100 vs. Control	74,00	100,0	-26,00	2,689	2	3	9,667	61,00
Sealer Plus 1/2 vs. Control	74,47	100,0	-25,53	2,689	2	3	9,493	61,00
Sealer Plus 1/4 vs. Control	77,18	100,0	-22,82	2,689	2	3	8,485	61,00
Sealer Plus 1/8 vs. Control	74,78	100,0	-25,22	2,406	3	3	10,48	61,00
Sealer 26 100 vs. Control	64,81	100,0	-35,19	2,406	3	3	14,63	61,00
Sealer 26 1/2 vs. Control	73,45	100,0	-26,55	2,406	3	3	11,04	61,00
Sealer 26 1/4 vs. Control	77,14	100,0	-22,86	2,406	3	3	9,503	61,00
Sealer 26 1/8 vs. Control	74,71	100,0	-25,29	2,406	3	3	10,51	61,00
Diaproseal 1/2 vs. Diaproseal 100	88,97	88,97	0,000	2,946	2	2	0,000	61,00
Diaproseal 1/4 vs. Diaproseal 100	94,11	88,97	5,142	2,689	3	2	1,912	61,00

Diaproseal 1/8 vs. Diaproseal 100	84,37	88,97	-4,598	2,689	3	2	1,710	61,00
Sealer Plus 100 vs. Diaproseal 100	74,00	88,97	-14,97	2,946	2	2	5,080	61,00
Sealer Plus 1/2 vs. Diaproseal 100	74,47	88,97	-14,50	2,946	2	2	4,920	61,00
Sealer Plus 1/4 vs. Diaproseal 100	77,18	88,97	-11,79	2,946	2	2	4,000	61,00
Sealer Plus 1/8 vs. Diaproseal 100	74,78	88,97	-14,19	2,689	3	2	5,274	61,00
Sealer 26 100 vs. Diaproseal 100	64,81	88,97	-24,16	2,689	3	2	8,981	61,00
Sealer 26 1/2 vs. Diaproseal 100	73,45	88,97	-15,52	2,689	3	2	5,770	61,00
Sealer 26 1/4 vs. Diaproseal 100	77,14	88,97	-11,83	2,689	3	2	4,397	61,00
Sealer 26 1/8 vs. Diaproseal 100	74,71	88,97	-14,26	2,689	3	2	5,302	61,00
Diaproseal 1/4 vs. Diaproseal 1/2	94,11	88,97	5,142	2,689	3	2	1,912	61,00
Diaproseal 1/8 vs. Diaproseal 1/2	84,37	88,97	-4,598	2,689	3	2	1,710	61,00
Sealer Plus 100 vs. Diaproseal 1/2	74,00	88,97	-14,97	2,946	2	2	5,080	61,00
Sealer Plus 1/2 vs. Diaproseal 1/2	74,47	88,97	-14,50	2,946	2	2	4,920	61,00
Sealer Plus 1/4 vs. Diaproseal 1/2	77,18	88,97	-11,79	2,946	2	2	4,000	61,00
Sealer Plus 1/8 vs. Diaproseal 1/2	74,78	88,97	-14,19	2,689	3	2	5,274	61,00
Sealer 26 100 vs. Diaproseal 1/2	64,81	88,97	-24,16	2,689	3	2	8,981	61,00
Sealer 26 1/2 vs. Diaproseal 1/2	73,45	88,97	-15,52	2,689	3	2	5,770	61,00
Sealer 26 1/4 vs. Diaproseal 1/2	77,14	88,97	-11,83	2,689	3	2	4,397	61,00
Sealer 26 1/8 vs. Diaproseal 1/2	74,71	88,97	-14,26	2,689	3	2	5,302	61,00
Diaproseal 1/8 vs. Diaproseal 1/4	84,37	94,11	-9,740	2,406	3	3	4,049	61,00
Sealer Plus 100 vs. Diaproseal 1/4	74,00	94,11	-20,11	2,689	2	3	7,476	61,00
Sealer Plus 1/2 vs. Diaproseal 1/4	74,47	94,11	-19,64	2,689	2	3	7,301	61,00
Sealer Plus 1/4 vs. Diaproseal 1/4	77,18	94,11	-16,93	2,689	2	3	6,294	61,00

Sealer Plus 1/8 vs. Diaproseal 1/4	74,78	94,11	-19,33	2,406	3	3	8,034	61,00
Sealer 26 100 vs. Diaproseal 1/4	64,81	94,11	-29,30	2,406	3	3	12,18	61,00
Sealer 26 1/2 vs. Diaproseal 1/4	73,45	94,11	-20,66	2,406	3	3	8,589	61,00
Sealer 26 1/4 vs. Diaproseal 1/4	77,14	94,11	-16,97	2,406	3	3	7,053	61,00
Sealer 26 1/8 vs. Diaproseal 1/4	74,71	94,11	-19,40	2,406	3	3	8,065	61,00
Sealer Plus 100 vs. Diaproseal 1/8	74,00	84,37	-10,37	2,689	2	3	3,855	61,00
Sealer Plus 1/2 vs. Diaproseal 1/8	74,47	84,37	-9,897	2,689	2	3	3,680	61,00
Sealer Plus 1/4 vs. Diaproseal 1/8	77,18	84,37	-7,187	2,689	2	3	2,672	61,00
Sealer Plus 1/8 vs. Diaproseal 1/8	74,78	84,37	-9,587	2,406	3	3	3,985	61,00
Sealer 26 100 vs. Diaproseal 1/8	64,81	84,37	-19,56	2,406	3	3	8,130	61,00
Sealer 26 1/2 vs. Diaproseal 1/8	73,45	84,37	-10,92	2,406	3	3	4,540	61,00
Sealer 26 1/4 vs. Diaproseal 1/8	77,14	84,37	-7,227	2,406	3	3	3,004	61,00
Sealer 26 1/8 vs. Diaproseal 1/8	74,71	84,37	-9,660	2,406	3	3	4,016	61,00
Sealer Plus 1/2 vs. Sealer Plus 100	74,47	74,00	0,4700	2,946	2	2	0,1595	61,00
Sealer Plus 1/4 vs. Sealer Plus 100	77,18	74,00	3,180	2,946	2	2	1,079	61,00
Sealer Plus 1/8 vs. Sealer Plus 100	74,78	74,00	0,7800	2,689	3	2	0,2900	61,00
Sealer 26 100 vs. Sealer Plus 100	64,81	74,00	-9,190	2,689	3	2	3,417	61,00
Sealer 26 1/2 vs. Sealer Plus 100	73,45	74,00	-0,5533	2,689	3	2	0,2057	61,00
Sealer 26 1/4 vs. Sealer Plus 100	77,14	74,00	3,140	2,689	3	2	1,168	61,00
Sealer 26 1/8 vs. Sealer Plus 100	74,71	74,00	0,7067	2,689	3	2	0,2628	61,00
Sealer Plus 1/4 vs. Sealer Plus 1/2	77,18	74,47	2,710	2,946	2	2	0,9198	61,00
Sealer Plus 1/8 vs. Sealer Plus 1/2	74,78	74,47	0,3100	2,689	3	2	0,1153	61,00
Sealer 26 100 vs. Sealer Plus 1/2	64,81	74,47	-9,660	2,689	3	2	3,592	61,00

Sealer 26 1/2 vs. Sealer Plus 1/2	73,45	74,47	-1,023	2,689	3	2	0,3805	61,00
Sealer 26 1/4 vs. Sealer Plus 1/2	77,14	74,47	2,670	2,689	3	2	0,9928	61,00
Sealer 26 1/8 vs. Sealer Plus 1/2	74,71	74,47	0,2367	2,689	3	2	0,08800	61,00
Sealer Plus 1/8 vs. Sealer Plus 1/4	74,78	77,18	-2,400	2,689	3	2	0,8924	61,00
Sealer 26 100 vs. Sealer Plus 1/4	64,81	77,18	-12,37	2,689	3	2	4,599	61,00
Sealer 26 1/2 vs. Sealer Plus 1/4	73,45	77,18	-3,733	2,689	3	2	1,388	61,00
Sealer 26 1/4 vs. Sealer Plus 1/4	77,14	77,18	-0,04000	2,689	3	2	0,01487	61,00
Sealer 26 1/8 vs. Sealer Plus 1/4	74,71	77,18	-2,473	2,689	3	2	0,9196	61,00
Sealer 26 100 vs. Sealer Plus 1/8	64,81	74,78	-9,970	2,406	3	3	4,145	61,00
Sealer 26 1/2 vs. Sealer Plus 1/8	73,45	74,78	-1,333	2,406	3	3	0,5543	61,00
Sealer 26 1/4 vs. Sealer Plus 1/8	77,14	74,78	2,360	2,406	3	3	0,9811	61,00
Sealer 26 1/8 vs. Sealer Plus 1/8	74,71	74,78	-0,07333	2,406	3	3	0,03049	61,00
Sealer 26 1/2 vs. Sealer 26 100	73,45	64,81	8,637	2,406	3	3	3,590	61,00
Sealer 26 1/4 vs. Sealer 26 100	77,14	64,81	12,33	2,406	3	3	5,126	61,00
Sealer 26 1/8 vs. Sealer 26 100	74,71	64,81	9,897	2,406	3	3	4,114	61,00
Sealer 26 1/4 vs. Sealer 26 1/2	77,14	73,45	3,693	2,406	3	3	1,535	61,00
Sealer 26 1/8 vs. Sealer 26 1/2	74,71	73,45	1,260	2,406	3	3	0,5238	61,00
Sealer 26 1/8 vs. Sealer 26 1/4	74,71	77,14	-2,433	2,406	3	3	1,012	61,00

24hs

Diaproseal 100 vs. Control	86,15	100,0	-13,85	2,406	3	3	5,758	61,00
Diaproseal 1/2 vs. Control	89,44	100,0	-10,56	2,406	3	3	4,391	61,00
Diaproseal 1/4 vs. Control	102,4	100,0	2,350	2,689	2	3	0,8738	61,00
Diaproseal 1/8 vs. Control	107,4	100,0	7,390	2,689	2	3	2,748	61,00
Sealer Plus 100 vs. Control	93,98	100,0	-6,023	2,406	3	3	2,504	61,00
Sealer Plus 1/2 vs. Control	101,1	100,0	1,097	2,406	3	3	0,4559	61,00
Sealer Plus 1/4 vs. Control	108,1	100,0	8,100	2,689	2	3	3,012	61,00
Sealer Plus 1/8 vs. Control	101,9	100,0	1,875	2,689	2	3	0,6972	61,00
Sealer 26 100 vs. Control	61,03	100,0	-38,97	2,689	2	3	14,49	61,00

Sealer 26 1/2 vs. Control	63,30	100,0	-36,70	2,406	3	3	15,26	61,00
Sealer 26 1/4 vs. Control	74,80	100,0	-25,20	2,406	3	3	10,47	61,00
Sealer 26 1/8 vs. Control	89,79	100,0	-10,21	2,689	2	3	3,796	61,00
Diaproseal 1/2 vs. Diaproseal 100	89,44	86,15	3,287	2,406	3	3	1,366	61,00
Diaproseal 1/4 vs. Diaproseal 100	102,4	86,15	16,20	2,689	2	3	6,024	61,00
Diaproseal 1/8 vs. Diaproseal 100	107,4	86,15	21,24	2,689	2	3	7,898	61,00
Sealer Plus 100 vs. Diaproseal 100	93,98	86,15	7,827	2,406	3	3	3,254	61,00
Sealer Plus 1/2 vs. Diaproseal 100	101,1	86,15	14,95	2,406	3	3	6,214	61,00
Sealer Plus 1/4 vs. Diaproseal 100	108,1	86,15	21,95	2,689	2	3	8,162	61,00
Sealer Plus 1/8 vs. Diaproseal 100	101,9	86,15	15,73	2,689	2	3	5,847	61,00
Sealer 26 100 vs. Diaproseal 100	61,03	86,15	-25,12	2,689	2	3	9,340	61,00
Sealer 26 1/2 vs. Diaproseal 100	63,30	86,15	-22,85	2,406	3	3	9,498	61,00
Sealer 26 1/4 vs. Diaproseal 100	74,80	86,15	-11,35	2,406	3	3	4,717	61,00
Sealer 26 1/8 vs. Diaproseal 100	89,79	86,15	3,640	2,689	2	3	1,353	61,00
Diaproseal 1/4 vs. Diaproseal 1/2	102,4	89,44	12,91	2,689	2	3	4,801	61,00
Diaproseal 1/8 vs. Diaproseal 1/2	107,4	89,44	17,95	2,689	2	3	6,675	61,00
Sealer Plus 100 vs. Diaproseal 1/2	93,98	89,44	4,540	2,406	3	3	1,887	61,00
Sealer Plus 1/2 vs. Diaproseal 1/2	101,1	89,44	11,66	2,406	3	3	4,847	61,00
Sealer Plus 1/4 vs. Diaproseal 1/2	108,1	89,44	18,66	2,689	2	3	6,939	61,00
Sealer Plus 1/8 vs. Diaproseal 1/2	101,9	89,44	12,44	2,689	2	3	4,625	61,00
Sealer 26 100 vs. Diaproseal 1/2	61,03	89,44	-28,41	2,689	2	3	10,56	61,00
Sealer 26 1/2 vs. Diaproseal 1/2	63,30	89,44	-26,13	2,406	3	3	10,86	61,00
Sealer 26 1/4 vs. Diaproseal 1/2	74,80	89,44	-14,63	2,406	3	3	6,083	61,00
Sealer 26 1/8 vs. Diaproseal 1/2	89,79	89,44	0,3533	2,689	2	3	0,1314	61,00
Diaproseal 1/8 vs. Diaproseal 1/4	107,4	102,4	5,040	2,946	2	2	1,711	61,00

Sealer Plus 100 vs. Diaproseal 1/4	93,98	102,4	-8,373	2,689	3	2	3,113	61,00
Sealer Plus 1/2 vs. Diaproseal 1/4	101,1	102,4	-1,253	2,689	3	2	0,4660	61,00
Sealer Plus 1/4 vs. Diaproseal 1/4	108,1	102,4	5,750	2,946	2	2	1,952	61,00
Sealer Plus 1/8 vs. Diaproseal 1/4	101,9	102,4	-0,4750	2,946	2	2	0,1612	61,00
Sealer 26 100 vs. Diaproseal 1/4	61,03	102,4	-41,32	2,946	2	2	14,03	61,00
Sealer 26 1/2 vs. Diaproseal 1/4	63,30	102,4	-39,05	2,689	3	2	14,52	61,00
Sealer 26 1/4 vs. Diaproseal 1/4	74,80	102,4	-27,55	2,689	3	2	10,24	61,00
Sealer 26 1/8 vs. Diaproseal 1/4	89,79	102,4	-12,56	2,946	2	2	4,263	61,00
Sealer Plus 100 vs. Diaproseal 1/8	93,98	107,4	-13,41	2,689	3	2	4,987	61,00
Sealer Plus 1/2 vs. Diaproseal 1/8	101,1	107,4	-6,293	2,689	3	2	2,340	61,00
Sealer Plus 1/4 vs. Diaproseal 1/8	108,1	107,4	0,7100	2,946	2	2	0,2410	61,00
Sealer Plus 1/8 vs. Diaproseal 1/8	101,9	107,4	-5,515	2,946	2	2	1,872	61,00
Sealer 26 100 vs. Diaproseal 1/8	61,03	107,4	-46,36	2,946	2	2	15,74	61,00
Sealer 26 1/2 vs. Diaproseal 1/8	63,30	107,4	-44,09	2,689	3	2	16,39	61,00
Sealer 26 1/4 vs. Diaproseal 1/8	74,80	107,4	-32,59	2,689	3	2	12,12	61,00
Sealer 26 1/8 vs. Diaproseal 1/8	89,79	107,4	-17,60	2,946	2	2	5,974	61,00
Sealer Plus 1/2 vs. Sealer Plus 100	101,1	93,98	7,120	2,406	3	3	2,960	61,00
Sealer Plus 1/4 vs. Sealer Plus 100	108,1	93,98	14,12	2,689	2	3	5,251	61,00
Sealer Plus 1/8 vs. Sealer Plus 100	101,9	93,98	7,898	2,689	2	3	2,937	61,00
Sealer 26 100 vs. Sealer Plus 100	61,03	93,98	-32,95	2,689	2	3	12,25	61,00
Sealer 26 1/2 vs. Sealer Plus 100	63,30	93,98	-30,67	2,406	3	3	12,75	61,00
Sealer 26 1/4 vs. Sealer Plus 100	74,80	93,98	-19,17	2,406	3	3	7,971	61,00
Sealer 26 1/8 vs. Sealer Plus 100	89,79	93,98	-4,187	2,689	2	3	1,557	61,00

Sealer Plus 1/4 vs. Sealer Plus 1/2	108,1	101,1	7,003	2,689	2	3	2,604	61,00
Sealer Plus 1/8 vs. Sealer Plus 1/2	101,9	101,1	0,7783	2,689	2	3	0,2894	61,00
Sealer 26 100 vs. Sealer Plus 1/2	61,03	101,1	-40,07	2,689	2	3	14,90	61,00
Sealer 26 1/2 vs. Sealer Plus 1/2	63,30	101,1	-37,79	2,406	3	3	15,71	61,00
Sealer 26 1/4 vs. Sealer Plus 1/2	74,80	101,1	-26,29	2,406	3	3	10,93	61,00
Sealer 26 1/8 vs. Sealer Plus 1/2	89,79	101,1	-11,31	2,689	2	3	4,204	61,00
Sealer Plus 1/8 vs. Sealer Plus 1/4	101,9	108,1	-6,225	2,946	2	2	2,113	61,00
Sealer 26 100 vs. Sealer Plus 1/4	61,03	108,1	-47,07	2,946	2	2	15,98	61,00
Sealer 26 1/2 vs. Sealer Plus 1/4	63,30	108,1	-44,80	2,689	3	2	16,66	61,00
Sealer 26 1/4 vs. Sealer Plus 1/4	74,80	108,1	-33,30	2,689	3	2	12,38	61,00
Sealer 26 1/8 vs. Sealer Plus 1/4	89,79	108,1	-18,31	2,946	2	2	6,215	61,00
Sealer 26 100 vs. Sealer Plus 1/8	61,03	101,9	-40,85	2,946	2	2	13,86	61,00
Sealer 26 1/2 vs. Sealer Plus 1/8	63,30	101,9	-38,57	2,689	3	2	14,34	61,00
Sealer 26 1/4 vs. Sealer Plus 1/8	74,80	101,9	-27,07	2,689	3	2	10,07	61,00
Sealer 26 1/8 vs. Sealer Plus 1/8	89,79	101,9	-12,09	2,946	2	2	4,102	61,00
Sealer 26 1/2 vs. Sealer 26 100	63,30	61,03	2,273	2,689	3	2	0,8453	61,00
Sealer 26 1/4 vs. Sealer 26 100	74,80	61,03	13,77	2,689	3	2	5,121	61,00
Sealer 26 1/8 vs. Sealer 26 100	89,79	61,03	28,76	2,946	2	2	9,762	61,00
Sealer 26 1/4 vs. Sealer 26 1/2	74,80	63,30	11,50	2,406	3	3	4,781	61,00
Sealer 26 1/8 vs. Sealer 26 1/2	89,79	63,30	26,49	2,689	2	3	9,848	61,00
Sealer 26 1/8 vs. Sealer 26 1/4	89,79	74,80	14,99	2,689	2	3	5,572	61,00

48hs

Diaproseal 100 vs. Control	72,82	100,0	-27,18	2,406	3	3	11,30	61,00
Diaproseal 1/2 vs. Control	78,51	100,0	-21,49	2,406	3	3	8,932	61,00
Diaproseal 1/4 vs. Control	93,19	100,0	-6,815	2,689	2	3	2,534	61,00

Diaproseal 1/8 vs. Control	109,7	100,0	9,745	2,689	2	3	3,623	61,00
Sealer Plus 100 vs. Control	75,88	100,0	-24,12	2,406	3	3	10,03	61,00
Sealer Plus 1/2 vs. Control	85,48	100,0	-14,52	2,406	3	3	6,038	61,00
Sealer Plus 1/4 vs. Control	96,63	100,0	-3,375	2,689	2	3	1,255	61,00
Sealer Plus 1/8 vs. Control	109,5	100,0	9,490	2,689	2	3	3,529	61,00
Sealer 26 100 vs. Control	16,90	100,0	-83,10	2,406	3	3	34,55	61,00
Sealer 26 1/2 vs. Control	28,24	100,0	-71,76	2,406	3	3	29,83	61,00
Sealer 26 1/4 vs. Control	34,40	100,0	-65,61	2,689	2	3	24,39	61,00
Sealer 26 1/8 vs. Control	73,44	100,0	-26,56	2,689	2	3	9,876	61,00
Diaproseal 1/2 vs. Diaproseal 100	78,51	72,82	5,690	2,406	3	3	2,365	61,00
Diaproseal 1/4 vs. Diaproseal 100	93,19	72,82	20,36	2,689	2	3	7,571	61,00
Diaproseal 1/8 vs. Diaproseal 100	109,7	72,82	36,92	2,689	2	3	13,73	61,00
Sealer Plus 100 vs. Diaproseal 100	75,88	72,82	3,060	2,406	3	3	1,272	61,00
Sealer Plus 1/2 vs. Diaproseal 100	85,48	72,82	12,65	2,406	3	3	5,260	61,00
Sealer Plus 1/4 vs. Diaproseal 100	96,63	72,82	23,80	2,689	2	3	8,850	61,00
Sealer Plus 1/8 vs. Diaproseal 100	109,5	72,82	36,67	2,689	2	3	13,63	61,00
Sealer 26 100 vs. Diaproseal 100	16,90	72,82	-55,92	2,406	3	3	23,25	61,00
Sealer 26 1/2 vs. Diaproseal 100	28,24	72,82	-44,58	2,406	3	3	18,53	61,00
Sealer 26 1/4 vs. Diaproseal 100	34,40	72,82	-38,43	2,689	2	3	14,29	61,00
Sealer 26 1/8 vs. Diaproseal 100	73,44	72,82	0,6167	2,689	2	3	0,2293	61,00
Diaproseal 1/4 vs. Diaproseal 1/2	93,19	78,51	14,67	2,689	2	3	5,455	61,00
Diaproseal 1/8 vs. Diaproseal 1/2	109,7	78,51	31,23	2,689	2	3	11,61	61,00
Sealer Plus 100 vs. Diaproseal 1/2	75,88	78,51	-2,630	2,406	3	3	1,093	61,00
Sealer Plus 1/2 vs. Diaproseal 1/2	85,48	78,51	6,963	2,406	3	3	2,895	61,00
Sealer Plus 1/4 vs. Diaproseal 1/2	96,63	78,51	18,11	2,689	2	3	6,734	61,00
Sealer Plus 1/8 vs. Diaproseal 1/2	109,5	78,51	30,98	2,689	2	3	11,52	61,00
Sealer 26 100 vs. Diaproseal 1/2	16,90	78,51	-61,61	2,406	3	3	25,61	61,00
Sealer 26 1/2 vs. Diaproseal 1/2	28,24	78,51	-50,27	2,406	3	3	20,90	61,00

Sealer 26 1/4 vs. Diaproseal 1/2	34,40	78,51	-44,12	2,689	2	3	16,40	61,00
Sealer 26 1/8 vs. Diaproseal 1/2	73,44	78,51	-5,073	2,689	2	3	1,886	61,00
Diaproseal 1/8 vs. Diaproseal 1/4	109,7	93,19	16,56	2,946	2	2	5,621	61,00
Sealer Plus 100 vs. Diaproseal 1/4	75,88	93,19	-17,30	2,689	3	2	6,433	61,00
Sealer Plus 1/2 vs. Diaproseal 1/4	85,48	93,19	-7,708	2,689	3	2	2,866	61,00
Sealer Plus 1/4 vs. Diaproseal 1/4	96,63	93,19	3,440	2,946	2	2	1,168	61,00
Sealer Plus 1/8 vs. Diaproseal 1/4	109,5	93,19	16,31	2,946	2	2	5,534	61,00
Sealer 26 100 vs. Diaproseal 1/4	16,90	93,19	-76,29	2,689	3	2	28,36	61,00
Sealer 26 1/2 vs. Diaproseal 1/4	28,24	93,19	-64,95	2,689	3	2	24,15	61,00
Sealer 26 1/4 vs. Diaproseal 1/4	34,40	93,19	-58,79	2,946	2	2	19,95	61,00
Sealer 26 1/8 vs. Diaproseal 1/4	73,44	93,19	-19,75	2,946	2	2	6,702	61,00
Sealer Plus 100 vs. Diaproseal 1/8	75,88	109,7	-33,86	2,689	3	2	12,59	61,00
Sealer Plus 1/2 vs. Diaproseal 1/8	85,48	109,7	-24,27	2,689	3	2	9,024	61,00
Sealer Plus 1/4 vs. Diaproseal 1/8	96,63	109,7	-13,12	2,946	2	2	4,453	61,00
Sealer Plus 1/8 vs. Diaproseal 1/8	109,5	109,7	-0,2550	2,946	2	2	0,08655	61,00
Sealer 26 100 vs. Diaproseal 1/8	16,90	109,7	-92,85	2,689	3	2	34,52	61,00
Sealer 26 1/2 vs. Diaproseal 1/8	28,24	109,7	-81,51	2,689	3	2	30,31	61,00
Sealer 26 1/4 vs. Diaproseal 1/8	34,40	109,7	-75,35	2,946	2	2	25,58	61,00
Sealer 26 1/8 vs. Diaproseal 1/8	73,44	109,7	-36,31	2,946	2	2	12,32	61,00
Sealer Plus 1/2 vs. Sealer Plus 100	85,48	75,88	9,593	2,406	3	3	3,988	61,00
Sealer Plus 1/4 vs. Sealer Plus 100	96,63	75,88	20,74	2,689	2	3	7,712	61,00
Sealer Plus 1/8 vs. Sealer Plus 100	109,5	75,88	33,61	2,689	2	3	12,50	61,00
Sealer 26 100 vs. Sealer Plus 100	16,90	75,88	-58,98	2,406	3	3	24,52	61,00

Sealer 26 1/2 vs. Sealer Plus 100	28,24	75,88	-47,64	2,406	3	3	19,81	61,00
Sealer 26 1/4 vs. Sealer Plus 100	34,40	75,88	-41,49	2,689	2	3	15,43	61,00
Sealer 26 1/8 vs. Sealer Plus 100	73,44	75,88	-2,443	2,689	2	3	0,9085	61,00
Sealer Plus 1/4 vs. Sealer Plus 1/2	96,63	85,48	11,15	2,689	2	3	4,145	61,00
Sealer Plus 1/8 vs. Sealer Plus 1/2	109,5	85,48	24,01	2,689	2	3	8,929	61,00
Sealer 26 100 vs. Sealer Plus 1/2	16,90	85,48	-68,58	2,406	3	3	28,51	61,00
Sealer 26 1/2 vs. Sealer Plus 1/2	28,24	85,48	-57,24	2,406	3	3	23,79	61,00
Sealer 26 1/4 vs. Sealer Plus 1/2	34,40	85,48	-51,08	2,689	2	3	18,99	61,00
Sealer 26 1/8 vs. Sealer Plus 1/2	73,44	85,48	-12,04	2,689	2	3	4,476	61,00
Sealer Plus 1/8 vs. Sealer Plus 1/4	109,5	96,63	12,87	2,946	2	2	4,367	61,00
Sealer 26 100 vs. Sealer Plus 1/4	16,90	96,63	-79,73	2,689	3	2	29,64	61,00
Sealer 26 1/2 vs. Sealer Plus 1/4	28,24	96,63	-68,39	2,689	3	2	25,43	61,00
Sealer 26 1/4 vs. Sealer Plus 1/4	34,40	96,63	-62,23	2,946	2	2	21,12	61,00
Sealer 26 1/8 vs. Sealer Plus 1/4	73,44	96,63	-23,19	2,946	2	2	7,870	61,00
Sealer 26 100 vs. Sealer Plus 1/8	16,90	109,5	-92,59	2,689	3	2	34,43	61,00
Sealer 26 1/2 vs. Sealer Plus 1/8	28,24	109,5	-81,25	2,689	3	2	30,21	61,00
Sealer 26 1/4 vs. Sealer Plus 1/8	34,40	109,5	-75,10	2,946	2	2	25,49	61,00
Sealer 26 1/8 vs. Sealer Plus 1/8	73,44	109,5	-36,05	2,946	2	2	12,24	61,00
Sealer 26 1/2 vs. Sealer 26 100	28,24	16,90	11,34	2,406	3	3	4,714	61,00
Sealer 26 1/4 vs. Sealer 26 100	34,40	16,90	17,50	2,689	2	3	6,505	61,00
Sealer 26 1/8 vs. Sealer 26 100	73,44	16,90	56,54	2,689	2	3	21,02	61,00
Sealer 26 1/4 vs. Sealer 26 1/2	34,40	28,24	6,155	2,689	2	3	2,289	61,00
Sealer 26 1/8 vs. Sealer 26 1/2	73,44	28,24	45,20	2,689	2	3	16,81	61,00

Sealer 26 1/8 vs. Sealer 26 1/4 73,44 34,40 39,05 2,946 2 2 13,25 61,00

Data analyzed: Data 1

Source of Variation	Degrees of Freedom	Sum of Squares	Mean square
Column Factor	12	27782	2315
Row Factor	2	4039	2020
Interaction	24	11889	495.4
Residual (error)	61	529.5	8.680
Total	99	44246	

Does Column Factor have the same effect at all values of Row Factor?
Interaction accounts for 26.87% of the total variance.
F = 57.07. DFn = 24, DFd = 61
The P value is < 0.0001
If there is no interaction overall, there is a less than 0.01% chance of randomly observing so much
interaction in an experiment of this size. The interaction is considered extremely significant.

Since the interaction is statistically significant, the P values that
follow for the row and column effects are difficult to interpret.

Does Column Factor affect the result?
Column Factor accounts for 62.79% of the total variance.
F = 266.73. DFn = 12, DFd = 61
The P value is < 0.0001
If Column Factor has no effect overall, there is a less than 0.01% chance of randomly observing an
effect this big (or bigger) in an experiment of this size. The effect is considered extremely significant.

Does Row Factor affect the result?
Row Factor accounts for 9.129% of the total variance.
F = 232.69. DFn = 2, DFd = 61
The P value is < 0.0001
If Row Factor has no effect overall, there is a less than 0.01% chance of randomly observing an
effect this big (or bigger) in an experiment of this size. The effect is considered extremely significant
