# Supplementary material for: Biological investigation of resinous endodontic sealers containing calcium hydroxide
Source: PLoS One. 2023 Jul 17;18(7):e0287890. doi: 10.1371/journal.pone.0287890 (PMC10351732; doi:10.1371/journal.pone.0287890)
Supplement: S2 File — Inhibitory halo induced by groups, measured by digital caliper. (PDF) [file pone.0287890.s002.pdf]

Antimicrobial Resinous sealer with calcium hydroxide

Petri dish 1	24 hours	48 hours
Dia Pro Seal	11.7	12.1
Sealer 26	13.7	14.6
Clorexidina	15.9	15.4
Sealer Plus	10.3	11.2

Petri dish 2	24 hours	48 hours
Dia Pro Seal	10.8	10.9
Sealer 26	17.3	21.2
Clorexidina	18.2	19.0
Sealer Plus	11.0	13.3

Petri dish 3	24 hours	48 hours
Dia Pro Seal	9.8	12.0
Sealer 26	13.2	13.6
Clorexidina	15.7	17.6
Sealer Plus	10.0	10.9

Petri dish 4	24 hours	48 hours
Dia Pro Seal	9.9	10.5
Sealer 26	16.7	16.8
Clorexidina	17.1	17.7
Sealer Plus	10.3	10.6

	Median 24hr	Median 48 hrs	24h Median -6mm	48h Median ·
Dia Pro Seal	10.5	11.3	4.5	5.3
Sealer 26	15.2	16.5	9.2	10.5
Clorexidina	16.7	17.4	10.7	11.4
Sealer Plus	10.4	11.5	4.4	5.5

Pesquisa: 12/07/2018
24 horas: 13/07/18
48 horas: 14/07/18
Paquímetro Digital Mitutoyo Absolute Digimatic, Japan, (mm)

h Median -6mm	x 0.5	x 0.5
	2.2	2.6
	4.6	5.2
	5.3	5.7
	2.2	2.7
