# Supplementary material for: Biological investigation of resinous endodontic sealers containing calcium hydroxide
Source: PLoS One. 2023 Jul 17;18(7):e0287890. doi: 10.1371/journal.pone.0287890 (PMC10351732; doi:10.1371/journal.pone.0287890)
Supplement: S3 File — Inflammatory score (1–3) induced by collected samples. (PDF) [file pone.0287890.s003.pdf]

	7 Dias	15 Dias	30 Dias
Controle	2	2	1
Controle	2	2	1
Controle	2	2	1
Controle	2	1	1
Controle	2	1	0
Controle	1	2	0
Controle	1	2	0
Controle	2	2	2
Controle	2	2	0
Controle	2	2	0
DiaProSeal	2	2	2
DiaProSeal	1	1	1
DiaProSeal	2	1	0
DiaProSeal	2	2	1
DiaProSeal	1	2	1
DiaProSeal	2	1	0
DiaProSeal	2	2	2
DiaProSeal	1	2	1
DiaProSeal	2	2	0
DiaProSeal	1	1	2
SealerPlus	2	2	2
SealerPlus	2	2	2
SealerPlus	1	2	1
SealerPlus	2	1	2
SealerPlus	2	2	0
SealerPlus	2	2	0
SealerPlus	1	1	2
SealerPlus	1	1	1
SealerPlus	2	1	1
SealerPlus	2	1	0
Sealer26	3	2	1
Sealer26	2	2	2
Sealer26	2	2	2
Sealer26	2	2	0
Sealer26	2	2	1
Sealer26	2	2	1
Sealer26	1	1	2
Sealer26	2	2	2
Sealer26	2	2	1
Sealer26	2	2	2

60 Dias
1
0
0
0
1
1
1
1
0
0
0
1
1
2
1
0
1
2
0
0
1
0
0
1
0
0
1
0
1
1
1
0
1
1
2
1
1
1
1
0
