# Supplementary material for: Biological investigation of resinous endodontic sealers containing calcium hydroxide
Source: PLoS One. 2023 Jul 17;18(7):e0287890. doi: 10.1371/journal.pone.0287890 (PMC10351732; doi:10.1371/journal.pone.0287890)
Supplement: S4 File — Red and Green fiber area in each group and final fiber percentage. (PDF) [file pone.0287890.s004.pdf]

	red fiber area	green fiber area	Total Area of Fibers	Final percentage of red fiber area, according to total fiber area
SP 7D Bl 2	3,49	27,77	31,26	11,16
SP 7D Bl 3	0,19	5,65	5,84	3,25
SP 7D Bl 4	1,11	2,23	3,34	33,23
SP 7D Bl 5	0,66	31,61	32,27	2,05
SP 7D Bl 6	0,01	3,11	3,12	0,32
SP 7D Bl 7	6,65	15,56	22,21	29,94
SP 15D Bl 1	8,16	11,18	19,34	42,19
SP 15D Bl 2	3,3	10,44	13,74	24,02
SP 15D Bl8	1,76	4,85	6,61	26,63
SP 15D Bl9	0,44	10,77	11,21	3,93
SP 30D Bl 1	17,23	18,3	35,53	48,49
SP 30D Bl 2	0,1	5,4	5,5	1,82
SP 30D Bl 4	3,11	21,47	24,58	12,65
SP 30D Bl 5	1,57	8,75	10,32	15,21
SP 30D Bl 6	6,29	19,86	26,15	24,05
SP 30D Bl 7	8,67	20,58	29,25	29,64
SP 30D Bl8	20,73	39,43	60,16	34,46
SP 30 D Bl9	4,5	15,4	19,9	22,61
SP 60 D Bl2	0,28	9,04	9,32	3,00
SP 60 D Bl3	0,9	5,71	6,61	13,62
SP 60 D Bl4	2,75	6,08	8,83	31,14
SP 60 D Bl5	7,18	15,35	22,53	31,87
SP 60 D Bl6	2,64	14,49	17,13	15,41
SP 60 D Bl7	2,66	6,99	9,65	27,56
SP 60 D Bl8	3,74	5,28	9,02	41,46
Dia 7D Bl 2	1,22	5,4	6,62	18,43
Dia 7D Bl 3	3,18	13,87	17,05	18,65
Dia 7D Bl 3A	6,3	13,24	19,54	32,24
Dia 7D Bl 5	1,15	4,38	5,53	20,80
Dia 7D Bl 7	2,07	4,87	6,94	29,83
Dia 7D Bl 9	0,01	3,79	3,8	0,26
Dia 15D Bl 1	0,61	4,16	4,77	12,79
Dia 15D Bl 2	1,58	7,12	8,7	18,16
Dia 15D Bl 3	2,85	4,33	7,18	39,69
Dia 15D Bl 4	0,4	2,03	2,43	16,46
Dia 15D Bl8	0,28	3,64	3,92	7,14
Dia 15D Bl10	1,62	2,51	4,13	39,23
Dia 30D Bl 1	0,99	7	7,99	12,39
Dia 30D Bl 2	3,31	12,25	15,56	21,27
Dia 30D Bl 4	3	5,76	8,76	34,25
Dia 30D Bl 7	0,21	3,71	3,92	5,36
Dia 30D Bl10	1,43	0,27	1,7	84,12
Dia 60 D Bl2	2,63	2,83	5,46	48,17
Dia 60 D Bl3	0,54	3,42	3,96	13,64
Dia 60 D Bl5	0,5	6,86	7,36	6,79

Dia 60 D Bl6	0,09	4,38	4,47	2,01
Dia 60 D Bl7	0,4	2,19	2,59	15,44
Dia 60 D Bl10	2,58	10,25	12,83	20,11
S26 7D Bl 1	3,62	3,04	6,66	54,35
S26 7D Bl 3	0,93	5,34	6,27	14,83
S26 7D Bl 6	0,77	2,79	3,56	21,63
S26 7D Bl 7	0,76	4,28	5,04	15,08
S26 7D Bl 8	0	2,22	2,22	0,00
S26 7D Bl 10	0	1,09	1,09	0,00
S26 15D Bl 2	0,76	1,71	2,47	30,77
S26 15D Bl 4	0,76	1,3	2,06	36,89
S26 15D Bl 5	0,41	4,35	4,76	8,61
S26 15D Bl 6	1,59	3,97	5,56	28,60
S26 15D Bl 7	0,3	1,18	1,48	20,27
S26 15D Bl9	3,69	3,28	6,97	52,94
S26 15D Bl10	0,16	0,47	0,63	25,40
S26 30D Bl 4	23,46	4,41	27,87	84,18
S26 30D Bl 6	5,44	2,85	8,29	65,62
S26 30D Bl9	3,53	5,24	8,77	40,25
S26 60 D Bl1	13,11	7,72	20,83	62,94
S26 60 D Bl2	23,03	13,57	36,6	62,92
S26 60 D Bl4	14	9,89	23,89	58,60
S26 60 D Bl5	4,91	9,62	14,53	33,79
S26 60 D Bl8	26,77	8,54	35,31	75,81

Final percentage of green fiber area, according to the total area of fibers
88,84
96,75
66,77
97,95
99,68
70,06
57,81
75,98
73,37
96,07
51,51
98,18
87,35
84,79
75,95
70,36
65,54
77,39
97,00
86,38
68,86
68,13
84,59
72,44
58,54
81,57
81,35
67,76
79,20
70,17
99,74
87,21
81,84
60,31
83,54
92,86
60,77
87,61
78,73
65,75
94,64
15,88
51,83
86,36
93,21

97,99
84,56
79,89
45,65
85,17
78,37
84,92
100,00
100,00
69,23
63,11
91,39
71,40
79,73
47,06
74,60
15,82
34,38
59,75
37,06
37,08
41,40
66,21
24,19

	Final percentage of red fiber area, according to total fiber area	Final percentage of green fiber area, according to the total area of fibers
SP 7D Bl 2	11,16	88,84
SP 7D Bl 3	3,25	96,75
SP 7D Bl 4	33,23	66,77
SP 7D Bl 5	2,05	97,95
SP 7D Bl 6	0,32	99,68
SP 7D Bl 7	29,94	70,06
SP 15D Bl 1	42,19	57,81
SP 15D Bl 2	24,02	75,98
SP 15D Bl8	26,63	73,37
SP 15D Bl9	3,93	96,07
SP 30D Bl 1	48,49	51,51
SP 30D Bl 2	1,82	98,18
SP 30D Bl 4	12,65	87,35
SP 30D Bl 5	15,21	84,79
SP 30D Bl 6	24,05	75,95
SP 30D Bl 7	29,64	70,36
SP 30D Bl8	34,46	65,54
SP 30 D Bl9	22,61	77,39
SP 60 D Bl2	3,00	97,00
SP 60 D Bl3	13,62	86,38
SP 60 D Bl4	31,14	68,86
SP 60 D Bl5	31,87	68,13
SP 60 D Bl6	15,41	84,59
SP 60 D Bl7	27,56	72,44
SP 60 D Bl8	41,46	58,54
Dia 7D Bl 2	18,43	81,57
Dia 7D Bl 3	18,65	81,35
Dia 7D Bl 3A	32,24	67,76
Dia 7D Bl 5	20,80	79,20
Dia 7D Bl 7	29,83	70,17
Dia 7D Bl 9	0,26	99,74
Dia 15D Bl 1	12,79	87,21
Dia 15D Bl 2	18,16	81,84
Dia 15D Bl 3	39,69	60,31
Dia 15D Bl 4	16,46	83,54
Dia 15D Bl8	7,14	92,86
Dia 15D Bl10	39,23	60,77
Dia 30D Bl 1	12,39	87,61
Dia 30D Bl 2	21,27	78,73
Dia 30D Bl 4	34,25	65,75
Dia 30D Bl 7	5,36	94,64
Dia 30D Bl10	84,12	15,88
Dia 60 D Bl2	48,17	51,83
Dia 60 D Bl3	13,64	86,36
Dia 60 D Bl5	6,79	93,21
Dia 60 D Bl6	2,01	97,99

Dia 60 D Bl7	15,44	84,56
Dia 60 D Bl10	20,11	79,89
S26 7D Bl 1	54,35	45,65
S26 7D Bl 3	14,83	85,17
S26 7D Bl 6	21,63	78,37
S26 7D Bl 7	15,08	84,92
S26 7D Bl 8	0,00	100,00
S26 7D Bl 10	0,00	100,00
S26 15D Bl 2	30,77	69,23
S26 15D Bl 4	36,89	63,11
S26 15D Bl 5	8,61	91,39
S26 15D Bl 6	28,60	71,40
S26 15D Bl 7	20,27	79,73
S26 15D Bl9	52,94	47,06
S26 15D Bl10	25,40	74,60
S26 30D Bl 4	84,18	15,82
S26 30D Bl 6	65,62	34,38
S26 30D Bl9	40,25	59,75
S26 60 D Bl1	62,94	37,06
S26 60 D Bl2	62,92	37,08
S26 60 D Bl4	58,60	41,40
S26 60 D Bl5	33,79	66,21
S26 60 D Bl8	75,81	24,19
